# Adaptive Network Stabilization Despite Profound Structural Reorganization in Human Organotypic Cultures

**DOI:** 10.1002/advs.77795

**Published:** 2026-09-16

**Authors:** Estilla Zsófia Tóth, Rebeka Stelcz, Réka Bod, Ábel Petik, Eszter Juhász, Kinga Tóth, Liza Szalai, Nour Essam, Fanni Somogyi, Beatrix Kovács, Amirmahdi Mojtahedzadeh, Attila György Bagó, Loránd Erőss, Péter Orbay, Johanna Petra Szabó, Dániel Fabó, Boglárka Hajnal, Bence Rácz, Daniel Hillier, István Ulbert, Lucia Wittner

**Affiliations:** ^1^ HUN‐REN Research Centre for Natural Sciences Budapest Hungary; ^2^ Szentágothai Doctoral School Semmelweis University Budapest Hungary; ^3^ Faculty of Information Technology and Bionics Péter Pázmány Catholic University Budapest Hungary; ^4^ Clinic for Neurosurgery and Neurointervention Semmelweis University Budapest Hungary; ^5^ Department of Anatomy and Histology University of Veterinary Medicine Budapest Budapest Hungary

**Keywords:** anatomy, calcium imaging, electrophysiology, human brain, neocortex, organotypic cultures

## Abstract

Human organotypic slice cultures provide experimental access to adult human neuronal circuits ex vivo, yet it remains unclear whether these networks preserve function or undergo fundamental reorganization following the profound perturbation of slice preparation. Here, a multimodal approach combines extracellular electrophysiology, longitudinal calcium imaging, and quantitative histology to track the changes of human cortical slice cultures over several weeks in vitro. Early phases are marked by pronounced variability and instability, with reduced firing rates, increased burst propensity in principal cells, and heterogeneous recruitment during population activity. These functional changes coincide with substantial structural remodeling, including considerable neuronal loss, disruption of laminar architecture, reactive gliosis, and selective vulnerability of inhibitory interneurons. Despite this structural degradation, neuronal activity converges: electrophysiological properties, cell‐type‐specific firing patterns, and population‐event recruitment become stable and highly consistent. Calcium imaging reveals the progressive evolution of spatially confined active regions, indicating the stabilization of structured network dynamics. These findings demonstrate a remarkable capacity of adult human neuronal circuits for functional adaptation, transitioning from heterogeneous, injury‐driven dynamics to stable and reproducible neuronal activity. This dissociation between anatomical deterioration and functional stabilization should be considered when using organotypic slice cultures as a platform for studying human physiological or pathological brain network dynamics.

## Graphical abstract

Adult human organotypic cortical slices undergo marked neuronal loss, laminar disruption, and glial activation, yet their network activity progressively stabilizes after a variable period. Electrophysiology, longitudinal calcium imaging, and quantitative histology show that structurally remodeled human circuits show adaptive capacity resulting in stable non‐epileptiform activity over five weeks in vitro, establishing a robust model for investigating mature human cortical network function and injury‐induced structural remodeling.

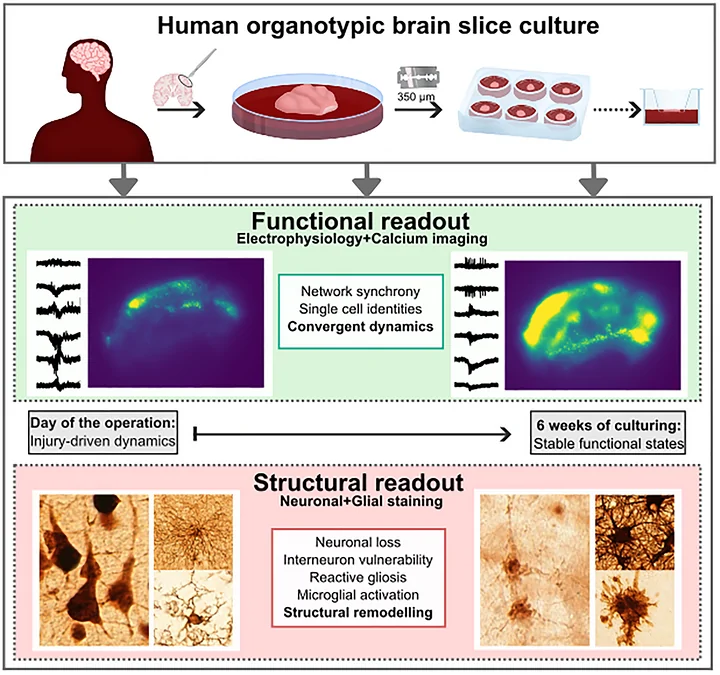

## 1 Introduction

Understanding how human neuronal circuits respond to injury‐related perturbations while providing a certain functional output remains a central question in neuroscience. The interpretation of brain activity ultimately depends on the underlying structural organization of neuronal and glial circuits. Although numerous neurological disorders exhibit a close correspondence between abnormal neuronal activity and structural pathology [1, 2, 3], others display profound functional/behavioural alterations despite relatively subtle anatomical abnormalities [4, 5], emphasizing that the relationship between brain structure and function is complex and might be poorly understood.

In vivo human studies provide limited access to cellular and circuit‐level mechanisms, whereas reductionist cell cultures lack the architectural and cellular complexity of intact brain networks. Acute slice preparations represent an excellent model to investigate human neuronal physiology [6, 7, 8, 9], genetics [10, 11, 12, 13, 14], and local synchrony [15, 16], but restrict the experimental window in time. Organotypic slice cultures bridge these gaps by preserving elements of native cytoarchitecture, local connectivity, and cell‐type diversity while enabling long‐term experimental access to neuronal circuits [17, 18, 19, 20, 21].

A critical distinction lies, however, between rodent and human organotypic models. Rodent slice cultures are typically derived from embryonic or early postnatal tissue, in which networks are still undergoing developmental maturation [22]. In contrast, human organotypic slices that are prepared from adult post‐mortem samples [20] or surgical resections [18, 23, 24, 25], often originate from epileptic or tumor‐associated cortex, and therefore represent fully developed and functionally specialized circuits with prior physiological and pathological history. This fundamental difference raises an important unresolved question: to what extent do adult human neuronal networks retain their intrinsic functional organization after the profound perturbation of surgery and slice preparation?

Studies in rodent organotypic cultures have established that slice preparation induces a cascade of network‐level changes, including dendritic and axonal transection, synaptic reorganization, and homeostatic plasticity, which together reshape neuronal activity over days to weeks in vitro [26]. These processes are typically associated with dynamic changes in firing rates, burst structure, and synchronization, reflecting the transition from an acutely deafferented state to a re‐equilibrated network. In human tissue, recent work has demonstrated the persistence of spontaneous and evoked activity in organotypic cultures [27, 28, 29], as well as the maintenance of certain cell‐type‐specific physiological properties [30]. However, a comprehensive understanding of how single‐neuron activity, population dynamics, and morphological properties co‐evolve over time in human slice cultures remains lacking.

Another unresolved issue concerns variability. Human surgical samples are inherently heterogeneous, reflecting differences in age, pathology, anatomical origin, and prior treatment. This variability poses a challenge for reproducibility but also offers an opportunity to assess whether intrinsic circuit mechanisms drive convergence toward stable functional states [31]. In rodent systems, network activity is strongly influenced by developmental stage and genetic background, often resulting in substantial variability across preparations [32]. Whether similar or distinct principles govern the evolution of human neuronal networks in vitro is not well understood.

To address these questions, we performed a multimodal analysis of human organotypic slice cultures, combining extracellular population and single‐unit electrophysiology, longitudinal calcium imaging, and quantitative histological assessment across extended culture periods. This approach enables simultaneous investigation of neuronal activity at multiple scales: from spike timing and cell‐type‐specific firing patterns to mesoscale population dynamics and underlying morphological organization. We hypothesized that the substantial structural perturbation predicted from the culturing methodology would profoundly affect the functional output. Instead, we found that substantial neuronal loss, laminar disorganization, interneuron vulnerability, and glial activation were accompanied by an early period of functional variability followed by the emergence of a stable neuronal activity highly resembling the acute state. These findings demonstrate that adult human circuits ex vivo exhibit a remarkable capacity for functional stabilization, cellular‐ and network‐level adaptation despite the extensive morphological reorganization. By providing a comprehensive characterization of this adaptive process, our study reveals a substantial dissociation between structural integrity and functional network dynamics, highlighting the flexibility of adult human cortical circuits.

## 2 Results

### 2.1 Organotypic Slice Cultures – Culturing Conditions

Organotypic cultures (HOC) were made from human neocortical samples obtained from *n* = 30 patients (Tables S1 and S2). These slices were kept alive for up to 42 days in vitro (D42). The viability and activity of the neurons in the cultures were investigated using different methods. In vitro electrophysiological recordings were made to verify neuronal activity both on the day of the operation (D0), and at different time points during the culturing period. In the case of cultured slices injected with adeno‐associated viruses (AAVs), neuronal activity was also assessed by wide‐field imaging. Furthermore, anatomical techniques were used to estimate the viability and the morphological changes of the slices: neuronal stainings NeuN, parvalbumin (PV) and calretinin (CR) showed the presence and the density of the surviving neurons, whereas glial activation was detected with astrocytic marker, glial fibrillary acidic protein (GFAP) and microglial marker, ionized calcium‐binding adapter molecule 1 (IBA1) stainings. This complex approach helped us to assess the relationship between neocortical activity and the survival of the neuronal and glial networks. Note that all slices used for electrophysiological recordings and Ca^2+^‐imaging were fixed and included in the quantitative anatomical study.

Slices derived from eleven patients were cultured in human cerebrospinal fluid (hCSF); slices obtained from 18 patients were kept in artificial medium (AM), whereas both hCSF and AM were tested in one tissue sample. During the first week of culturing, slices were treated with penicillin and streptomycin (PS) or PS completed with amphotericin B (PSA) to prevent bacterial and fungal infections. Due to a suboptimal hCSF collection method, the quality of the hCSF was highly unstable (see Supporting Information, Methods section). This fact was considered when drawing conclusions from the results.

### 2.2 In Vitro Electrophysiological Recordings

**FIGURE 1:**
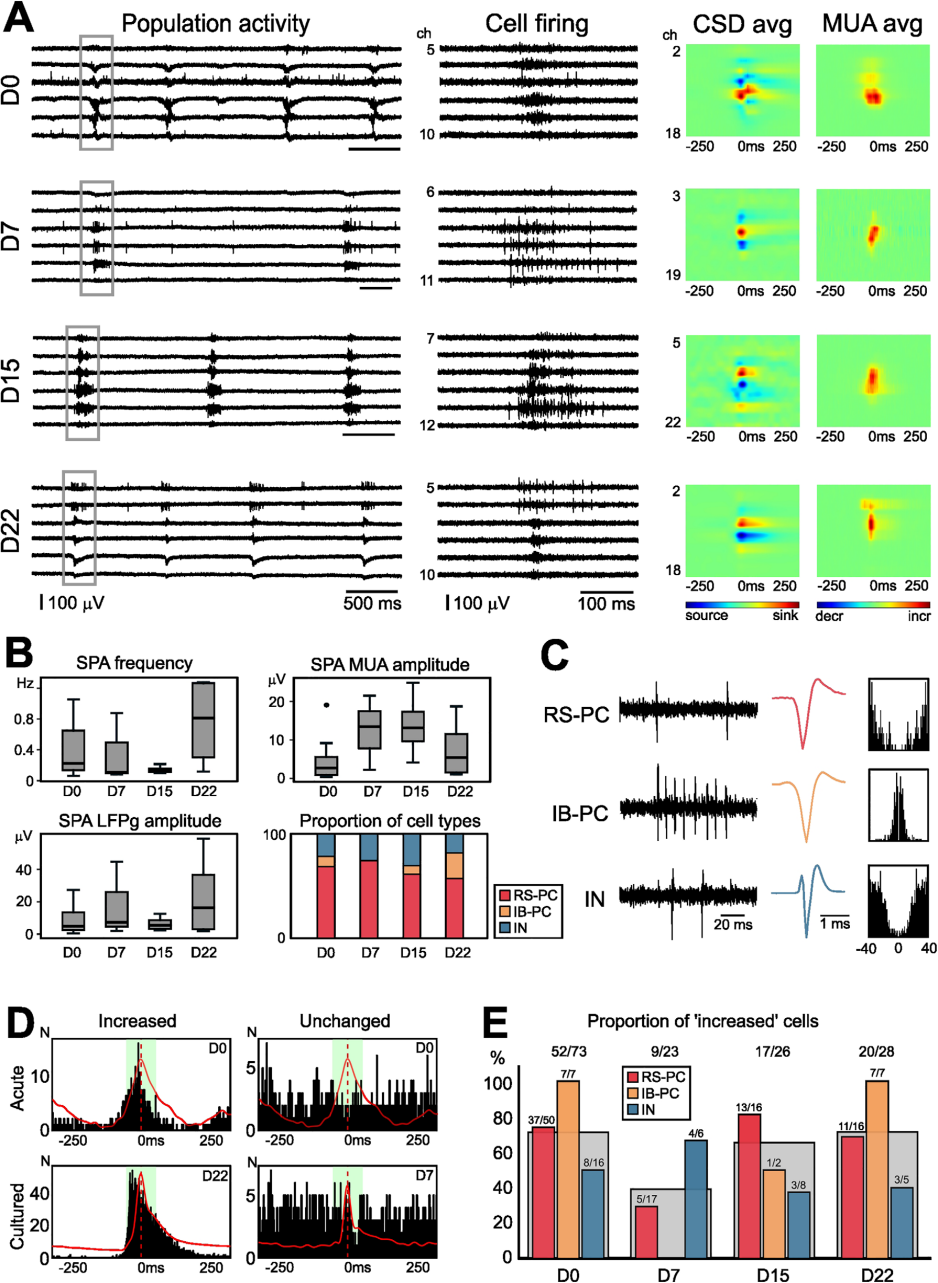
Multiple‐channel electrophysiological recordings of organotypic cultures. (A) Broad‐band recordings (left panel) show the spontaneous population activity (SPA) generated in human acute (D0) and cultured (D7, D15, D22) slices. SPA events (grey boxes) consisted of a local field potential transient with increased cell firing. Heat maps generated from current source density (CSD) and multi‐unit activity (MUA) analyses show that a complex sink‐source pattern and increased cell firing characterized the events. On the CSD heat maps, sinks are marked with warm colors, and sources with cold colors. On the MUA maps, warm colors represent MUA increase. Heat maps were generated from the time‐averaged (avg) SPA. Firing of single neurons (Cell firing) is shown in high‐pass‐filtered (500 Hz) recordings in the second panel from the left. (B) Box plots show the recurrence frequency (left top panel), the local field potential gradient (LFPg) amplitude (left bottom), and the MUA amplitude (right top) of SPAs on different days of the culturing period. The proportion of the different cell types among the clustered neurons is shown in the bottom‐right panel. RS‐PC: regular spiking principal cell, IB‐PC: intrinsically bursting principal cell, IN: inhibitory interneuron. (C) High‐pass (>500) filtered recordings (left panels), average action potential shape generated from broad‐band recording (middle panel), and autocorrelogram of one example of RS‐PC (top), IB‐PC (middle), and IN (bottom) are shown. (D) Peri‐event time histograms demonstrate that clustered single neurons showed either increased or unchanged firing rate during SPA events, both in acute and cultured slices. Red lines show the averaged LFPg of the respective SPA, whereas the green shading marks the duration of the SPA (±50 ms) taken into account in the analysis. (E) Proportion of neurons with increased firing rate during the SPAs during the culturing period. Red bars depict RS‐PCs, orange bars mark IB‐PCs, whereas INs are marked with blue bars. All cells pooled are shown by grey bars.

Both acute slices and organotypic slice cultures were investigated by in vitro electrophysiological methods. Extracellular recordings were made using a 24‐channel linear microelectrode [33] placed perpendicular to the pial surface to record from the entire width of the cortex. Altogether 90 slices from 17 patients were included in the electrophysiological analysis (D0: 62 slices in 15 patients, D7: 9 slices in 7 patients, D15: 5 slices in 5 patients, D22: 9 slices in 7 patients, D30: 4 slices in 4 patients and D35: 1 slice). Cellular firing as well as the presence of spontaneous population activity [34] (SPA) emerging in standard physiological baths were noted in all slices (Figure 1). In the acute samples (D0) cell firing could be detected in 73% of the slices; 37% of them generated SPA as well. On D7, organotypic slices displayed cellular firing and SPA in 56% and 44%, respectively. Neuronal firing was observed in all D14 slices, of which 60% also produced SPA. On D21, 78% and 33% of slices displayed cellular firing and SPA, respectively. On D30, neuronal firing could be detected in all slices, and 50% of them generated SPA as well; finally, the D35 slice showed cell firing without the emergence of SPA. In nine HOC samples, we recorded both on D0 and in cultured slices. In five cases, SPA was recorded both in acute (D0) and in cultured slices. In three cases, SPA could be detected only on D0, but not in the cultures, and in one case, SPA was not present on D0 but emerged in all of the cultured slices.

The recurrence frequency of SPAs in the acute slices (at D0, *n* = 11 SPAs from 6 slices from 3 patients, Figure 1B) was 0.38 ± 0.34 Hz (mean±st.dev.). The recurrence frequency did not change during the culturing period, ranging from 0.05 Hz (D30) to 1.28 ± 0.76 Hz (D22, Kruskal‐Wallis ANOVA, *p* = 0.3238, 95% confidence interval, CI: [0.213, 0.559], ε^2^ = 0.099; see Table S3). The local field potential gradient (LFPg) amplitude was 94.1 ± 92.1 µV at D0 and was not significantly different during the culturing period (Kruskal‐Wallis ANOVA, *p* = 0.9051, CI: [56.4, 188], ε^2^ = 0.027). The CSD and the MUA amplitudes were also unchanged across all different days examined (Kruskal‐Wallis ANOVA, *p* = 0.9679 and *p* = 0.1129, ε^2^ = 0.012 and 0.284, respectively). The CSD was 79 ± 67.5 µV at D0 and ranged between 34.6 µV (D30) and 189 ± 213 µV (D22), with a 95% CI [53, 155]. The MUA was 4.59±5.63 µV at D0 and ranged between 4.12 µV (D30) to 13.9±8.63 µV (D15), 95% CI [4.45, 11].

A total of 151 single units (neurons) were clustered in *n* = 17 recordings, where SPA was detected (from *n* = 4 patients, Figure 1C). In the acute slices (4 slices from 3 patients, at D0) a total of 73 neurons were clustered: 57 principal cells (PC, 50 regular spiking, RS, 7 intrinsically bursting, IB) and 16 inhibitory interneurons (IN). In two slices (from 2 patients) recorded at D7, a total of 23 cells were identified: 17 PCs (all RS) and 6 INs. In two slices (from two patients) at D15, we clustered 26 neurons: 18 PCs (16 RS + 2 IB) and 8 INs. In two slices from two patients at D22, 16 RS‐PCs, 7 IB‐PCs, and 5 INs gave a total of 28 cells, whereas we could cluster one IN at D30 (in one slice). The ratio of interneurons did not significantly change during the culturing time (from D0 to D22); it varied between 18 and 31%.

To examine the cellular properties of the clustered neurons (see Methods), we determined the average firing rate, the maximal firing rate, burstiness index 1 (ratio of action potentials, APs within burst, where burst contains at least 3 APs, is preceded and followed by a 20 ms silent period, and where any three consecutive APs fall within 20 ms), burstiness index 2 (the percentage of ISI values less than 20 ms), half‐width 1 (half‐width of the largest amplitude of the AP) and half‐width 2 (half‐width of the second peak, which gives an estimation on the afterhyperpolarization part of the AP), interspike interval coefficient of variation (ISI CV, giving an estimate about the wide or narrow distribution of the ISI values). Higher ISI CV values indicate more irregular firing than lower values.

The average firing rate of all neurons (from D0 to D30) was 1.55 ± 1.92 Hz, CI = [1.24, 1.86]. The firing rate was similar in the different cell groups (PCs vs. INs, as well as IB‐PC vs. RS‐PC vs. IN; see Supporting Information). The average firing rate of all neurons was quite variable along the culturing period: it was significantly higher at D7 and significantly lower at D15 compared to D0 and D22 (see Table S4). To examine the effects of culturing on neuronal cellular features, we compared the different cell types clustered in acute slices with those in organotypic cultures (from D7 to D22, pooled). We found changes in several cellular properties affecting different cell types. The firing rate of RS‐PCs (but not that of IB‐PCs or INs) decreased significantly in culture. The burstiness index 1 of IB‐PCs (but not that of RS‐PCs and INs) significantly increased in culturing conditions. Note that both burstiness indices were significantly higher for IB‐PCs than the two other cell types, and burstiness index 1 of IB‐PCs further increased in culture. Consistent with these groupwise effects, rank‐based trend analyses showed a modest monotonic tendency across days‐in‐vitro for IB‐PCs (burstiness ISI < 20 ms, *ρ* = 0.18). Interestingly, the half‐width 1 of RS‐PCs (but not that of IB‐PCs and INs) decreased significantly in culture compared to acute slices. RS‐PC spike half‐width 1 exhibited a clear negative trend with culture time (*ρ* = −0.48), consistent with progressive waveform narrowing within this principal cell population. At the same time, half‐width 2 did not change for any of the examined cell types. The ISI CV significantly increased for RS‐PCs and INs, but not for IB‐PCs in culture. When we examined all days, we detected a modest trend in the change in ISI CV (*ρ* = −0.23, for more details, see Supporting Information).

Next, we verified how the clustered neurons were involved in the population events (SPA) detected in the slice. Therefore, we determined for each cell whether it increased its firing rate during SPA events, with the aid of an adapted Monte‐Carlo approach [15] (see Methods). We could not detect any cells with decreased firing rate, only with increased or unchanged firing rate (Figure 1B,D,E). In the acute slices, 53/73 cells (71%) increased their firing rate, whereas the others remained unchanged. The ratio of participating cells (with increased firing rate) was significantly different during the culturing period: 9/23 (39%) cells at D7, 17/26 (65%) neurons at D15, and 20/28 (71%) cells at D22 increased their firing rate during SPA events (Chi‐square test, *p* = 0.009). Regarding the involvement of the different cell types, we found that all three neuron groups participated in the generation of SPAs, although at significantly different ratios (67% of RS‐PCs, 94% of IB‐PCs, and 50% of INs; Chi‐square test, *p* = 0.036, Figure 1). Within‐cell‐type comparisons indicated that responsive (“increased”) RS‐PCs had higher ISI < 20 ms burstiness than non‐responsive RS‐PCs (*p* < 0.001), whereas responsive interneurons fired faster (*p* = 0.022) and were less bursty (*p* = 0.040) than INs with unchanged firing rate.

### 2.3 Wide‐Field Imaging

To assess neuronal activity in the organotypic slice cultures, we performed wide‐field imaging of slices expressing GCaMP following viral transduction (Methods, Supporting Information). Seventeen slices (from three patients) were recorded across D1 to D31 post‐injection. Neuronal GCaMP expression was confirmed in 15 of 15 injected slices, whereas two non‐injected slices served as negative controls and showed no fluorescent transients.

**FIGURE 2:**
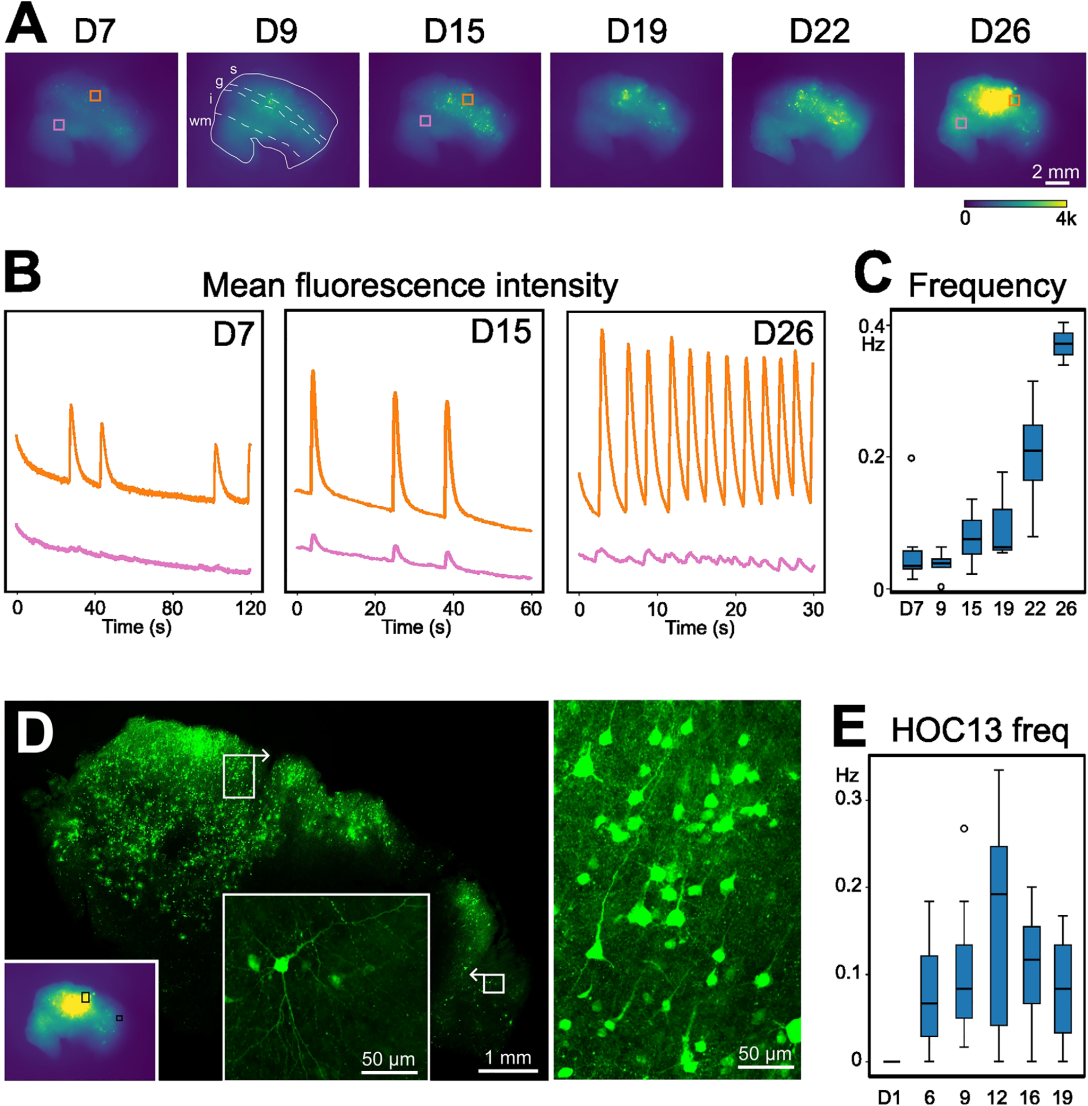
Wide‐field calcium imaging reveals spatially confined SPA over time in virally transduced human organotypic slices. (A) Time‐averaged fluorescence intensity images of a single organotypic slice (HOC15_p1w3, undiluted virus) at different time points of the culturing period (from D7 to D26). The slice was injected with EF1a‐GCaMP6s‐WPRE (PHP.eB, titer: 2.47E+13 vg/mL) and fixed on D28. Warmer colors (green‐yellow) show higher fluorescence (arbitrary units, 0–4000); the same color scale applies to all images. Contours show the different layers of the neocortex: s: supragranular (L1‐3), g: granular (L4), i: infragranular (L5‐6), wm: white matter. (B) Fluorescence intensity traces (arbitrary units) from two spatially distinct regions of interest (orange and pink squares in A) at D7, D15, and D26. Peaks correspond to population Ca^2+^‐transients. SPA events are not consistently synchronous between regions, indicating multiple hubs of activity generation. Note that time axes differ between recording days and are indicated below each trace. (C) Ca^2+^‐transient recurrence frequency measured over the full field of view on the slice in (A) across the culturing period. Consistent with the traces shown in (B), the frequency gradually increases with time spent in culture. (D) Confocal image of the slice shown in (A) and (B) demonstrates the presence of numerous GCaMP‐positive cells. Numerous cells are present all over the whole slice, although with variable density. Boxed regions (upper, lower) are shown at higher magnification in the right panel and inset, respectively. The locations of the insets are shown on the time‐averaged fluorescence intensity image (black boxes). (E) Ca^2+^‐transient recurrence frequency of SPA across the culturing period for all slices (*n* = 6) from one HOC sample. Note that prior to GCaMP expression, at D1, zero frequency reflects reporter absence. Consistent detection of transients across slices from D6 onward indicates reliable reporter expression and recurrent SPA with stable frequency over the culture period.

GCaMP signal was first detected on D4 in slices receiving undiluted virus and remained recordable until D31. Across the 14 slices with longitudinal recordings, fluorescence intensity and spatial extent increased over time in the majority of cases (10/14 slices, Figure 2; Figure S2A,D,G).

Population calcium activity – the correlate of SPA recorded with electrophysiology – was quantified by GCaMP‐reported transients over the full slice. All 15 GCaMP‐positive slices showed circumscribed regions of synchronous Ca^2+^ fluctuations on at least one recording day, and negative controls showed none. These active spots usually remained spatially stable during the culturing time and could be observed at every imaging occasion, up to D31. GCaMP‐related activity appeared first at D4 (*n* = 2/6 slices) and was sustained in 11 of 15 slices (73%) for at least 19 days post‐injection. The temporal profiles of Ca^2^^+^‐transient frequency varied across slices and across patients. The frequency gradually increased with time in most slices (*n* = 5, Figure 2B; Figure S2A), only one slice showed a decreasing pattern. In two slices, the frequency was low in the early period (up to D9), then stayed high until the end of the culturing time. A further three slices showed a biphasic pattern: increasing frequency to D9‐D20, which declined at the end (D22‐D31) of the culturing period (see Figure S2E). In three slices from the same patient, the recurrence frequency of the population events was stable over the entire culturing time (see Figure S2H).

Based on native slice images and post hoc anatomy, we identified the location of the GCaMP expression in all 15 virus‐containing slices (Figure S3). In most slices, GCaMP+ cells were visible in supragranular and infragranular layers (*n* = 7 slices); in two slices, GCaMP expression was confined only to supragranular layers; in two further slices, to supragranular+granular layers; and in one slice to granular+infragranular layers. Three additional slices showed GCaMP expression in all three layers. In slices recorded on at least three different days (*n* = 14 slices) we determined the location of the active regions as well. In the majority of slices (10/14), synchronous Ca^2^^+^ fluctuations originated from a single large active region. This region either remained spatially stable throughout the culture period (*n* = 4), expanded within the same layer (*n* = 2), or extended into adjacent layers (*n* = 4). In the remaining four slices, multiple small, spatially distinct active regions were present at the beginning of the culturing period, which progressively merged into a single large active region at later stages of the culture period.

The spatial extent of the active region closely corresponded to the distribution of GCaMP expression in all slices except one, in which GCaMP expression expanded over time while the active region remained spatially confined.

### 2.4 Quantitative Morphological Analysis

To characterize the morphological changes in the human neocortical organotypic cultures, we performed quantitative histological analysis on 280 slices derived from 28 patients (Table S2), using NeuN staining to assess neuronal density, GFAP and IBA1 to estimate astroglial and microglial activation, respectively, as well as parvalbumin (PV) and calretinin (CR) immunolabeling to quantify inhibitory interneuron subtypes. Culturing produced consistent and substantial structural remodeling: neuronal density considerably declined, both inhibitory interneuron subtypes were significantly reduced, and reactive gliosis was prominent throughout the culture period. Unless otherwise stated, data from epileptic and tumor‐associated samples were pooled where no significant group difference was found. Effects of culturing conditions — medium type (human cerebrospinal fluid, hCSF vs. artificial medium, AM), antimicrobial supplement (penicillin+streptomycin, PS vs. PS+amphotericinB, PSA), and viral treatment — are reported separately where applicable. One human organotypic culture (HOC) sample was considered as the sum of the slices made from a tissue sample derived from one patient, including both acute slices (D0) and cultured slices (from D1 up to D42). We hypothesized that virus injection affects the morphological status of the organotypic slices. Therefore, slices injected with viruses were treated separately and were considered as separate HOC samples, even if they derived from the same patient.

#### 2.4.1 Neuronal Loss in Human Organotypic Cultures

**FIGURE 3:**
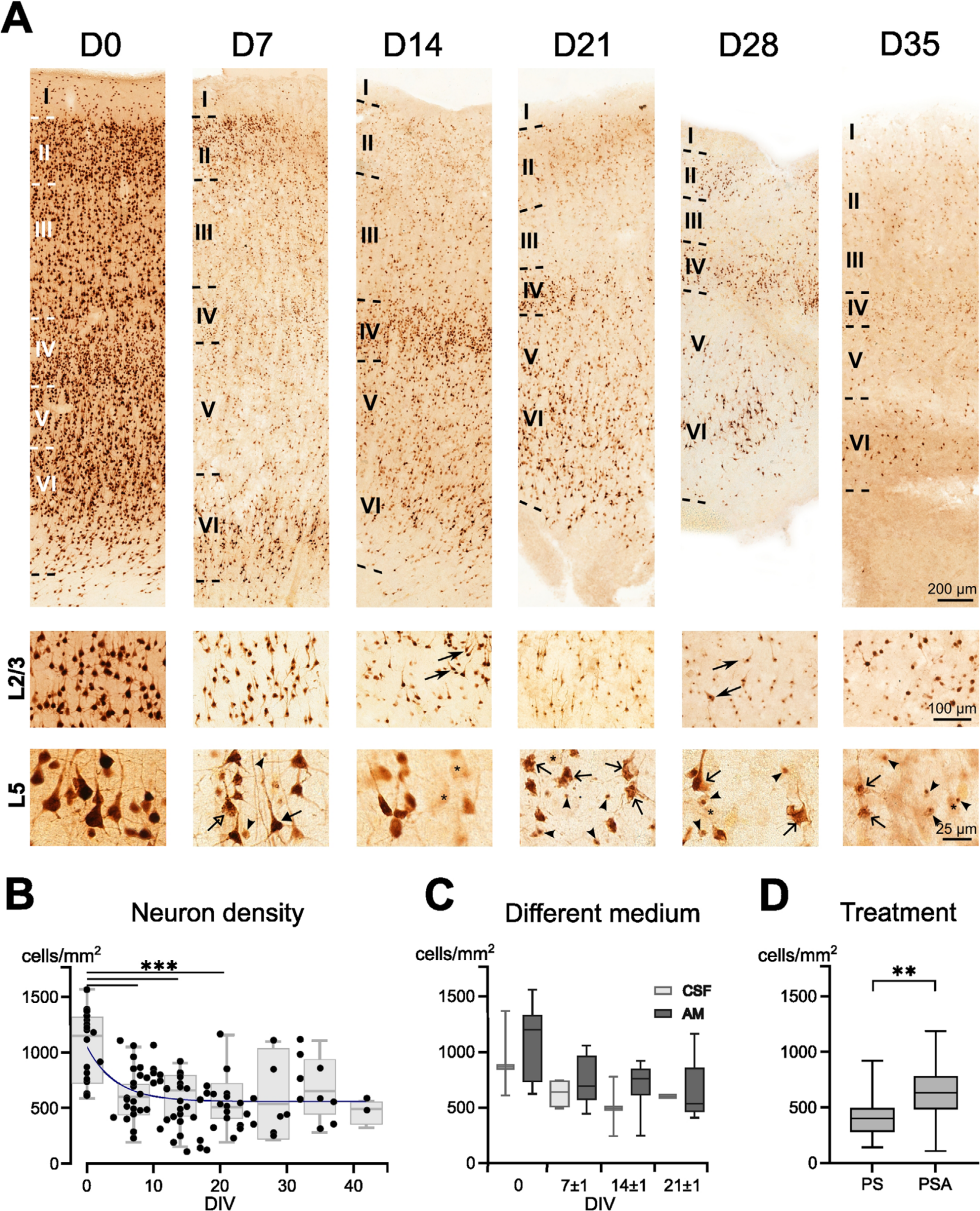
Considerable neuronal death occurs in organotypic cultures. (A) The cortical structure of human organotypic slices is preserved during the culturing period. The upper row of pictures shows the entire cortex, including all visible layers; the middle row shows the neuron density at higher magnification, whereas in the bottom row the morphology of the neurons can be seen. The human neocortex shows the typical 6‐layered structure in acute slices (D0) in NeuN‐stained sections. At D7, all 6 layers can be distinguished, but after the second week (from D14), layer borders usually vanish due to neuron loss. At D0, neocortical neurons show normal morphology: the nucleus is dark, and NeuN is visible in the proximal dendrites (middle and bottom). After one week of culture, the background of the NeuN‐stained section starts to clear up, but most neurons still show normal morphology (arrow on bottom image). However, cells with inhomogeneous NeuN‐staining (triangle arrow) and NeuN‐positive cellular fragments (open arrow) appear. In several cases, the surviving neurons were organized in columns (left side of D7 bottom image). After the considerable cell loss of the first week, the orientation of the surviving neurons was often changed; the apical dendrite of the pyramidal cells arose in different directions (sharp‐edged arrows on the D14 middle image). Furthermore, numerous neurons were only lightly stained (asterisks on bottom images), and more neuronal fragments, debris (arrowheads on bottom images), and neurons with distorted cell bodies and dendrites (open arrows on bottom images) could be detected. (B) Neuronal density shows a significant decrease during the first two weeks of culture, then it stays stable. Black dots represent the neuron density of each examined slice, fixed at different time points (*n* = 109), whereas grey boxplots show data averaged at the end of each week (±1 day; n(acute) = 17, n(D7) = 13, n(D14) = 16, n(D21) = 9, n(D28) = 4, n(35) = 8, n(42) = 4). Neuron density at the end of the first, second, and third weeks was significantly different from D0 (ANOVA, ^***^ *p* = 0.0006). (C) No significant differences (unpaired *t*‐test) were found when comparing neuron densities in slices kept either in human cerebrospinal fluid (hCSF, n(acute) = 3, n(D7) = 4, n(D14) = 3, n(D21) = 1) or in artificial medium (AM, n(acute) = 11, n(D7) = 7, n(D14) = 10, n(D21) = 7). However, note that cell densities are higher in AM. (D) Antibiotic treatment (Penicillin+Streptomycin, PS) resulted in significantly lower neuron densities than antibiotic+antifungal (PS+amphotericinB, PSA) treatment (unpaired *t*‐test, ^**^ *p* = 0.0075, n(PS) = 13, n(PSA) = 16).

The acute neocortical samples (at D0) showed the typical 6‐layered structure of the human neocortex in all cases. Compared to D0, the laminar architecture of the neocortex has been notably changed during culturing (Figure 3). The neuronal density visibly decreased over time, although the most pronounced cell loss occurred during the first week. While all six neocortical layers were visible in the acute tissue, after D7 the layer borders started to vanish. Intracellular modifications were also observed over time. Compared to the more homogeneous staining, NeuN was mainly localized within the nucleus, and the cytoplasm became lightly labeled (Figure 3). Neuronal debris appeared in the neuropil already after the first week. These changes occurred earlier when slices were cultured in hCSF. The use of AM resulted in better preservation of the neurons and the cortical structure, a decrease in neuronal cell numbers, and the appearance of debris (Figure S4). Neuronal survival was variable during the time course of the culturing period: large differences were seen in the neuronal density and in the integrity of the cells (lack of NeuN‐positive debris), which could affect either supragranular or infragranular or both layers. The background of the sections became considerably brighter with time; however, this change was also very variable among samples.

We performed cell counting on NeuN‐positive sections of *n* = 118 organotypic slices, ranging from D4 to D42, plus *n* = 17 D0 slices, derived from a total of 25 patients (for exact numbers of slices see Table S2, for cell density values see Table 1 and Table S9). There were no significant differences between the examined 34 HOC samples (28 control samples, 6 virally injected samples; Kruskal‐Wallis ANOVA, *p* = 0.0805). No differences were seen when comparing epileptic to non‐epileptic samples at D0 (unpaired t‐test, *p* = 0.1506) and thus, the data derived from epileptic and tumor patients were pooled for further analyses. The cell density significantly decreased over time in samples without viral injections (day‐by‐day changes, Supporting material, Kruskal‐Wallis ANOVA, *p* = 0.0105, Figure 3B). Significantly reduced cell density was observed when examined at the end of every week (week‐by‐week analysis, see Table S9), i.e., at D7±1, D14±1 and D21±1 compared to D0 (ANOVA, *p* = 0.0006).

**TABLE 1:** Quantitative histological data, week‐by‐week changes. Neuron densities (cells/mm^2^) are shown for NeuN, PV, and CR stainings. Glial coverage (%) is given for GFAP and IBA1 stainings. Furthermore, the perimeter‐to‐area ratio (PAR) is also given for IBA1+ cells. All data are given as mean ± standard deviation and median [first quartile, third quartile]. Note the large differences between the mean and median.

		D0		D7 ± 2		D14 ± 2		D21 ± 2		D28 ± 2		D33+	
		n	value	n	value	n	value	n	value	n	value	n	value
NeuN density (cells/mm^2^)	Mean ± st.dev	17	1068 ± 333	22	640 ± 234	28	598 ± 248	19	599 ± 320	9	590 ± 341	13	621 ± 399
	Median [Q1 Q3]		1116 [761 1291]		602 [488 789]		567 [414 815]		536 [392 690]		442 [327 804]		585 [357 697]
GFAP coverage (%)	Mean ± st.dev	20	39.35 ± 17.84	19	23.34 ± 16.51	29	32.78 ± 15.95	13	36.47 ± 20.60	7	26.88 ± 13.28	12	34.87 ± 16.98
	Median [Q1 Q3]		36.98 [25.85 52.65]		19.53 [10.81 33.43]		30.41 [22.52 44.92]		41.47 [19.52 54.20]		27.24 [17.30 29.25]		36.62 [21.37 43.33]
IBA1 coverage (%)	Mean ± st.dev	11	29.72 ± 23.50	5	16.12 ± 7.78	9	16.44 ± 9.76	7	21.38 ± 8,64	1	21.04	9	37.99 ± 18.95
	Median [Q1 Q3]		19.22 [13.78 38.39]		17.15 [11.14 17.65]		11.47 [9.18 28.26]		25.55 [13.52 27.68]		21.04		34.04 [26.27 40.74]
IBA1 PAR	Mean ± st.dev	11	2.09 ± 0.37	5	3.86 ± 0.80	9	3.96 ± 0.89	7	3.90 ± 0.22	1	4.04	9	3.43 ± 0.36
	Median [Q1 Q3]		1.94 [1.82 2.37]		4.07 [3.59 4.10]		4.03 [3.89 4.55]		3.93 [3.70 4.06]		4.04		3.52 [3.09 3.62]
PV density (cells/mm^2^)	Mean ± st.dev	14	32 ± 24	9	12 ± 14	11	6 ± 10	7	7 ± 9	4	13 ± 7	14	4 ± 6
	Median [Q1 Q3]		25 [19 34]		2 [1 20]		2 [0 6]		6 [0 8]		14 [9 18]		2 [0 6]
CR density (cells/mm^2^)	Mean ± st.dev	11	70 ± 46	7	34 ± 38	10	26 ± 22	8	14 ± 11	4	11 ± 9	8	18 ± 20
	Median [Q1 Q3]		69 [39 92]		27 [5 48]		18 [10 35]		12 [4 23]		9 [7 13]		8 [2 37]

No statistical difference was observed when we examined slices with or without viral infection derived from the same patients (Wilcoxon test, *p* = 0.9344). Despite the considerable differences in neuronal morphology and in the cortical laminarization changes in slices kept in hCSF or AM (see Figure S4 and Table S9), we could not show significant differences in neuronal density (Figure 3C, unpaired t‐test, *p* = 0.1285). The use of antibacterial PS treatment resulted in significantly lower values in neuronal density than in slices with complex antibacterial+antifungal PSA treatment (Figure 3D, unpaired *t*‐test, *p* = 0.0075).

#### 2.4.2 Astroglial Activation

**FIGURE 4:**
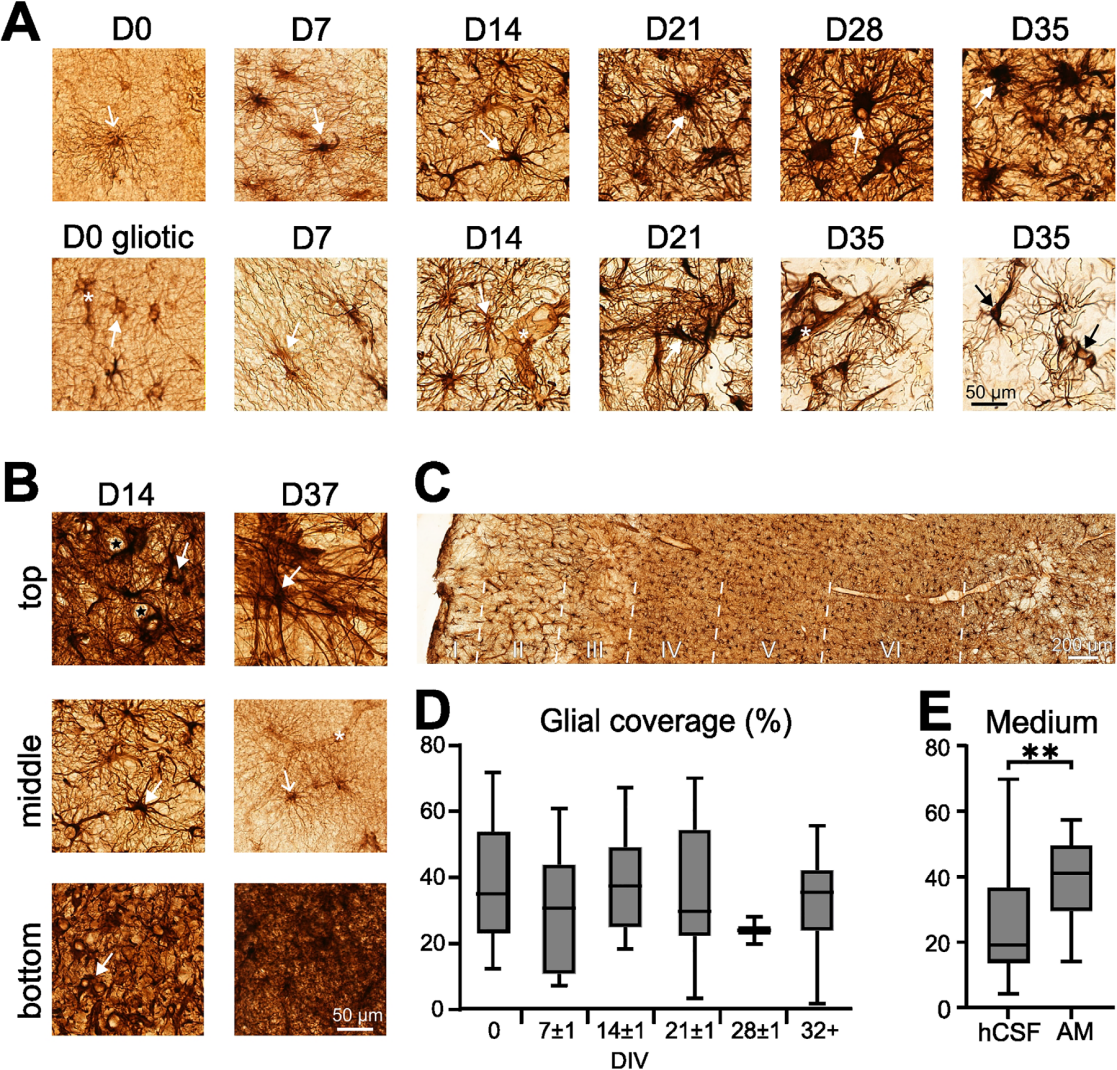
GFAP‐positive astroglial cells are highly activated in organotypic cultures. (A) Morphology of GFAP+ astroglial cells during the course of the culture period. In the acute tissue, GFAP+ cells showed either the morphology of resting astroglial cells (D0, open arrow) or signs of gliosis (D0 gliotic), where GFAP+ activated astroglial cells possessed larger cell bodies and thickened processes (arrow), both types forming the typical endfeet around the blood vessels (asterisks). During the culture period, activated astroglial cells were visible in all cases; resting astrocytes were very rarely seen. GFAP staining was quite variable; it became either very dark with large, dark cell bodies and numerous thick and heavily stained processes (arrows on upper row of images), or the background became clear and GFAP+ activated astroglial cells showed a less dense distribution (bottom row of images), with numerous thin processes. Blood vessels (asterisks) were usually drawn by the astroglial endfeet, even at D35. (B) A heavily stained glial scar was formed on the two surfaces of the slices (top and bottom), whereas moderate (D14) or little (D37) gliosis was observed in the middle of the slices. Arrows point to activated astroglial cells; an open arrow shows a resting glial cell (D37, middle), whereas an asterisk marks a long blood vessel present at D37. Empty holes (black stars, D14 top) or long glial processes (D37 top) were present in some cases on the top of the slices. Note that the GFAP staining is the strongest on the bottom of the slices. (C) GFAP‐staining delineates the layers of the neocortex in a D14 organotypic culture. A very dense and dark network covers the granular (L4) and infragranular layers (L5‐6), whereas activated astrocytes form a looser network in the supragranular layers (L1‐3) and in the white matter. (D) Glial coverage did not significantly change during the culturing period. Box plots show average data at the end of each week (±1 day, n(acute) = 20, n(D7) = 12, n(D14) = 15, n(D21) = 7, n(D28) = 2, n(35) = 11). Note that the glial coverage showed high variability among the samples throughout the culturing period. (E) Significantly lower glial coverage was seen in slices kept in hCSF compared to AM (unpaired t‐test, ^**^ *p* = 0.0045, n(CSF) = 19, n(AM) = 18).

To examine the changes in the astroglial network and to reveal the formation of a possible glial scar, we performed glial fibrillary acidic protein (GFAP) staining on *n* = 104 organotypic slices plus 20 samples fixed at D0, derived from 25 patients (Table 1; Table S9). In about half (11 of 20) of the acute slices (D0), the neocortical GFAP+ cells showed the features of resting astroglial cells. In the remaining samples, gliosis was observed, and activated astroglial cells were visible either in patches or extending to the entire slice. Gliosis was not related to epilepsy, as it occurred in samples derived from both epileptic (*n* = 4) and non‐epileptic tumor (*n* = 5) patients. Six out of 20 slices were also used for electrophysiological recording, and four of them were gliotic. During the culturing period, we detected a dramatic change in the astroglial morphology. Astroglial cells became very heavily stained, the size of their cell body increased, and their processes became thick (Figure 4). Blood vessel endfeet were usually strongly stained, although the vessels were most likely not functional in organotypic slices (see Figure S5). Different astroglial patterns were observed in the cultures. Either scattered glial cells were visible with a very bright, clean background, or a very dense glial meshwork covered part or the entire area of the slice. Reorganization of the GFAP+ network within the 3D structure of the organotypic slice showed large differences: a glial scar formed on the top and the bottom of the slice, whereas in the middle only moderate gliosis was observed (Figure 4). In several cases, we saw long glial processes stretching from layer 1 into the deeper layers, where a sparse network of glial cells was visible. This phenomenon was most prominent in virally injected slices, but was also visible in non‐injected cultures as well (Figure S5F).

Gliosis was quantified by determining glial coverage within the slices (Methods, Table 1; Table S9). In general, glial coverage showed high variability between sections. Glial coverage was not significantly different between the HOC samples (Kruskal‐Wallis ANOVA, *p* = 0.1316), nor between slices treated either with PS or with PSA (unpaired *t*‐test, *p* = 0.9718). Despite the striking modifications in the morphology of the astroglial cells, glial coverage did not differ during the culturing period (day‐by‐day changes, Kruskal‐Wallis test, *p* = 0.1644), nor when we compared the end of each week to D0 (ANOVA, *p* = 0.5611). Slices kept in hCSF showed lower glial coverage than those cultured in AM (unpaired *t*‐test, *p* = 0.0045), but viral injection had no effect on the glial coverage (Wilcoxon test, *p* = 0.3125).

#### 2.4.3 Microglial Activation and Morphological Heterogeneity

**FIGURE 5:**
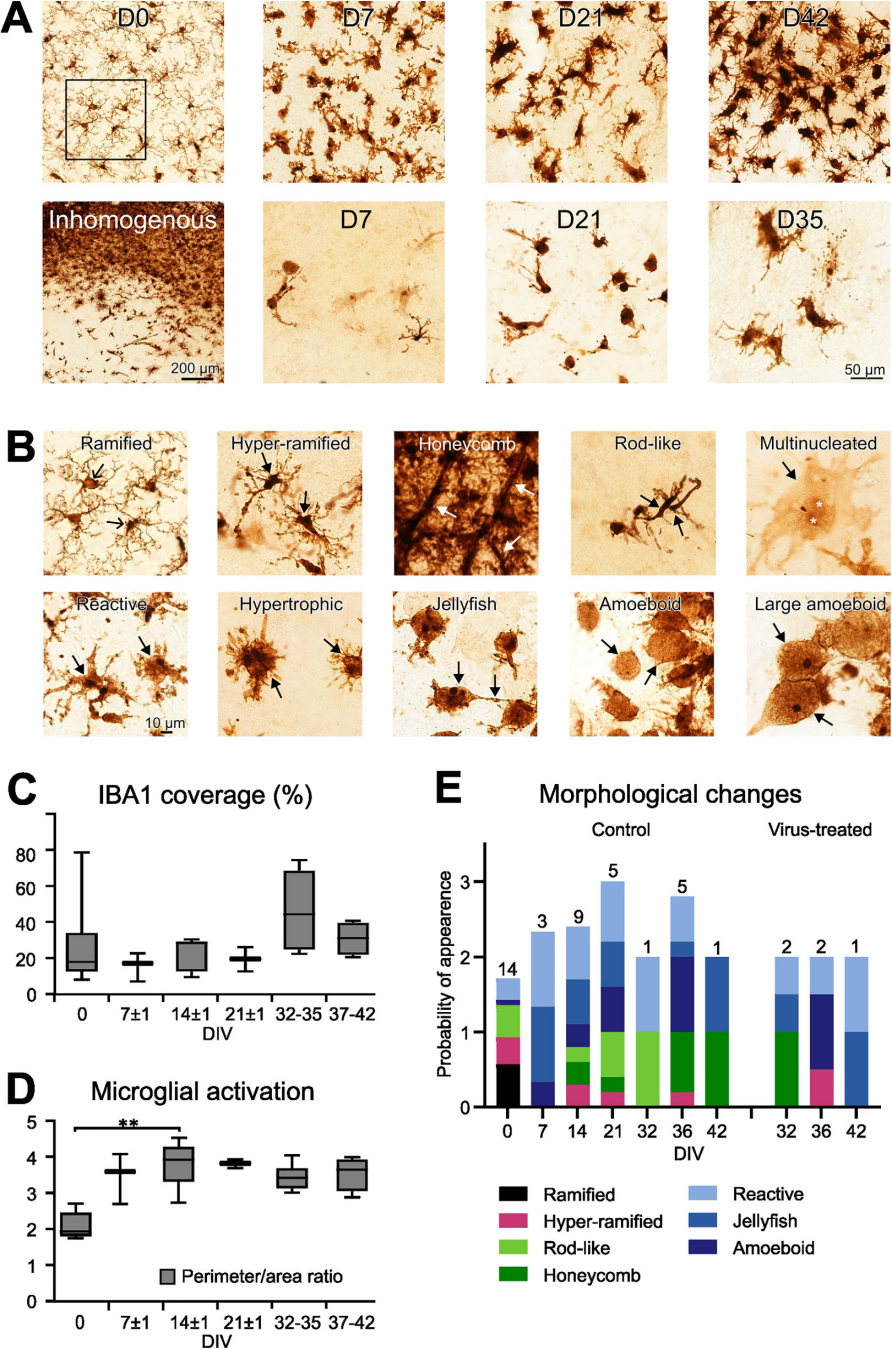
IBA1‐positive microglial cells show a dramatic change in human neocortical organotypic cultures. (A) Morphology and distribution of IBA1+ microglial cells. At D0, resting microglial cells were visible. After D0, only activated microglial cells were observed; resting IBA1+ cells disappeared. Areas with high (upper row) and low (lower row) density of microglial cells were both present during the entire culturing time (D7 to D42). Patches of high‐ and low‐density IBA1‐stained areas could be detected next to each other, in the same slice (Inhomogeneous). (B) Microglial cells showed very variable morphology. Resting cells have ramified morphology (open arrows), i.e., a small cell body and several ramifying processes. Black arrows show all different types of activated glial cells in the images. Hyperramified cells have small cell bodies with numerous thin processes. Honeycomb structures are composed of several cells with large and dark processes (white arrows). Rod‐like cells have small and elongated cell bodies and only a few (if any) processes. We have seen multinucleated cells with giant cell bodies (white asterisks show the nuclei). Usually, these cells are lightly stained. Reactive microglial cells have large cell bodies with several thick processes. Hypertrophic cells possess very large cell bodies and numerous short processes, whereas jellyfish‐type microglia usually have only a few short processes. Amoeboid and large amoeboid cells have round giant cell bodies without processes. Note that the same scale bar applies to all images. (C) Microglial coverage of the organotypic slices did not change significantly during the culturing period (Kruskal‐Wallis ANOVA, *p* = 0.3015, n(acute) = 11, n(D7) = 3, n(D14) = 5, n(D21) = 3, n(D32‐35) = 8, n(37‐42) = 4). Note the large variability of the IBA1 coverage at D0. DIV: days in vitro. (D) The perimeter/area ratio showed that significant microglial activation occurs during the first 2 weeks. The perimeter/area doubled compared to D0 and remained high during the culture period. (Kruskal‐Wallis ANOVA, ^**^ *p* = 0.0029, n(acute) = 11, n(D7) = 3, n(D14) = 5, n(D21) = 3, n(D32‐35) = 8, n(37‐42) = 4). (E) Probability of appearance of the different types of microglial cells during the culture period. The ratio of slices containing the different microglial types was determined at each examined day (DIV) and added. The numbers of examined slices are shown above the bars. Virus‐injected slices (right) were examined separately. Ramified microglial cells (black) were found only at D0, in about half of the slices. Stress‐linked hyper‐ramified types (magenta) were seen relatively rarely. Injury‐related honeycomb and rod‐like microglia (green bars) were seen from the second week. The phagocytic types (amoeboid, jellyfish, and reactive, blue bars) were present during the entire culture period.

The morphology of microglial cells is linked to their functions. A ramified appearance is characteristic of the homeostatic resting state when they surveil the neighboring neurons. Rod‐like and honeycomb morphologies suggest injury‐induced activation; hyper‐ramified forms appear in stress, whereas reactive, jellyfish, and ball‐like amoeboid types are known to show a phagocytic function [35, 36, 37]. To assess the changes in the microglial network in organotypic cultures, IBA1 staining was performed on a total of *n* = 40 organotypic slices plus 10 samples fixed at D0, derived from a total of 13 patients (Table 1; Table S9). In almost half of the acute samples (D0), IBA1‐positive microglial cells were distributed homogeneously and showed the homeostatic ramified morphology (*n* = 4/10 samples). In the remaining six cases, activated microglial morphologies were seen, such as hyper‐ramified (*n* = 4/6 samples), reactive (*n* = 2/6), and elongated amoeboid‐like (*n* = 1/5) microglial cells. Rod‐like microglia were visible together with homeostatic, ramified cells in two cases, whereas they were present with other activated cell types in six cases. All sections containing homeostatic ramified microglial cells were derived from therapy‐resistant epileptic patients, whereas the samples with activated microglial cells were derived mainly from patients with tumors (5 tumor patients, 1 epileptic patient). In the cultured slices, drastic morphological changes occurred. The distribution of the microglial cells was inhomogeneous, both spatially (Figure 5A) and morphologically (Figure 5B). The large differences in the density of the IBA1+ cells—such as in the case of GFAP+ astroglial cells—were also linked to the top‐to‐bottom gradient of the slice (Figure S6), i.e., dense networks of activated microglia were present at the surface of the slice, whereas the density of microglial cells was lower in the middle of the slice. The appearance of the IBA1+ cells was extremely variable within and among the slices and patients. The size of the cell body ranged from small to giant; the number of processes ranged from zero to very high numbers. Furthermore, the thickness and the length of their processes were also considerably variable, as was the intensity of their IBA1‐staining, in all different combinations of all the above features. In culture, homeostatic resting types totally disappeared, while all other types were detected. The ratio of the slices showing rod‐like, honeycomb (related to injury), hyper‐ramified (linked to stress), and ball‐like amoeboid, jellyfish, and reactive‐hypertrophic types (related to phagocytosis) was determined during the course of the culturing period (Figure 5E). Rod‐like and hyper‐ramified forms were usually seen in the earlier phase of the culture period (D7‐D21), but the appearance of these types could be associated with viral injection at later stages (D32‐D37) as well. Honeycomb microglia appeared mainly at later phases, both in virally injected and non‐injected slices (D32‐D42). Phagocytic, reactive, jellyfish, and amoeboid were the most frequently seen forms throughout the entire culturing period. In several cases, different forms of microglial cells, such as amoeboid and reactive types, formed large groups and were located next to each other (Figure S6A–C). In organotypic slices kept in hCSF, amoeboid/phagocytic forms were more frequently seen, whereas in AM, more microglial cells showed stress‐related activation, i.e, having smaller cell bodies and numerous long, thin processes (hyper‐ramified type).

To quantify changes in microglial activation during the culture period, we determined IBA1 coverage (percentage of the examined slice area) and calculated the perimeter‐to‐area ratio (PAR) of IBA1+ cells within predefined areas (see Methods, Table 1; Table S9). Despite the considerable morphological changes over the culturing period, microglial coverage did not show significant modifications (Kruskal‐Wallis ANOVA, *p* = 0.3015). Furthermore, we could not demonstrate significant differences between the tissue samples derived from different patients (*n* = 13 samples without viral treatment, *n* = 37 slices, Kruskal‐Wallis test, *p* = 0.3566), nor when comparing different culture conditions (hCSF vs AM: Mann‐Whitney test, *p* = 0.6620; viral treatment vs. not: paired t‐test, *p* = 0.9296). However, the difference in microglial coverage between the middle and the surface of the slices increased to 6‐fold over 6 weeks of culturing, as also shown by the increase in standard deviation (see Supporting Information).

In accordance with the coverage, the PAR did not show significant differences between the cultures (Kruskal‐Wallis test, *p* = 0.2438) nor over the culturing period (Kruskal‐Wallis test, *p* = 0.5553) nor when slices were treated with viruses (Wilcoxon signed rank test, *p* = 0.25). However, the week‐by‐week changes were significantly different (Kruskal‐Wallis ANOVA, *p* = 0.0029). After the second week, the activation level of microglial cells—shown by the PAR—significantly differed from the original state (multiple comparison Dunn's test, *p* = 0.0047). In AM, the PAR was significantly lower compared to hCSF (Mann‐Whitney test, *p* = 0.0027).

#### 2.4.4 Loss of Parvalbumin‐Positive Perisomatic Inhibitory Cells

**FIGURE 6:**
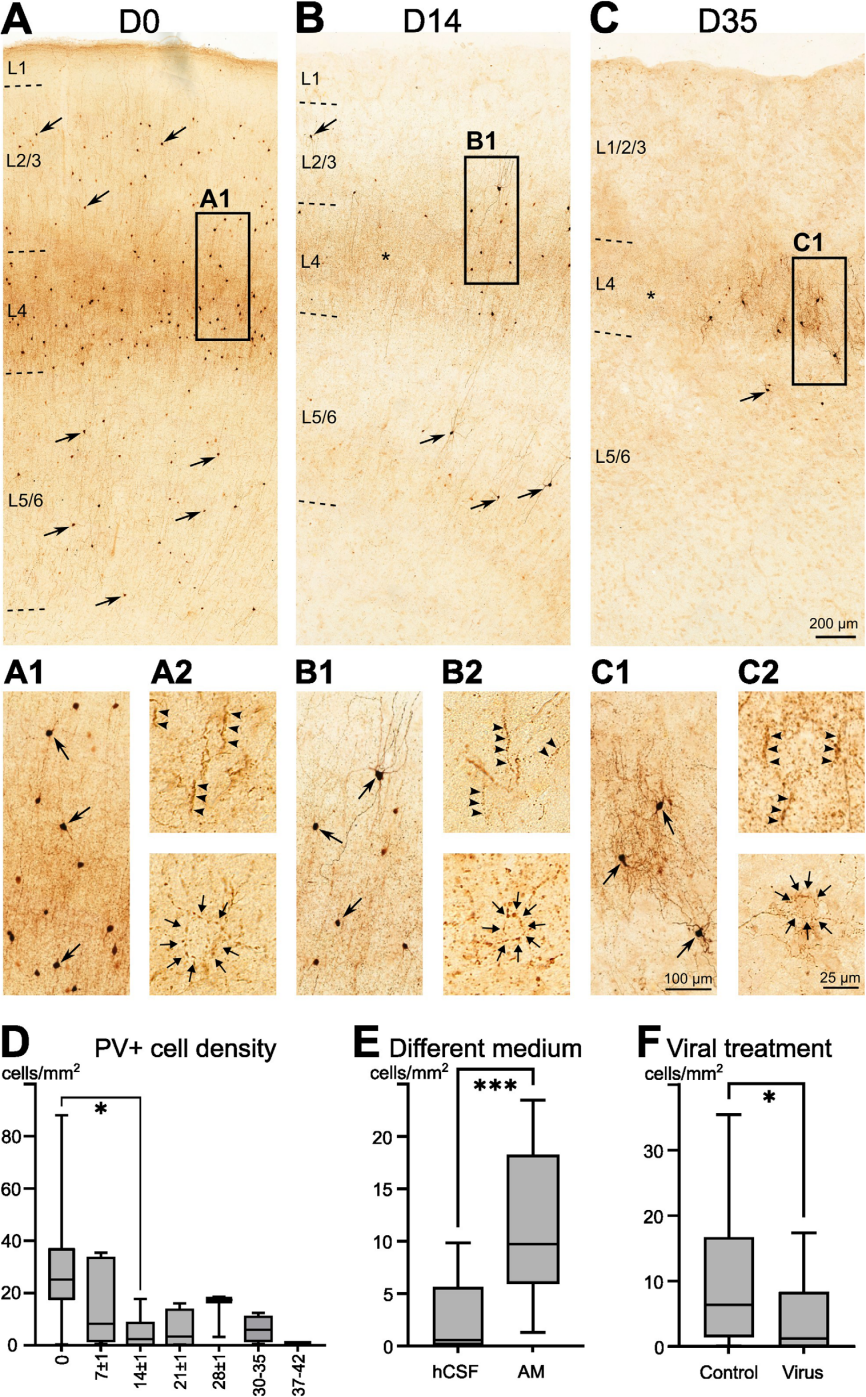
PV‐stained cells are mainly lost in organotypic cultures, but the remaining ones preserve their functions. (A) Parvalbumin‐stained section from an acute sample. Numerous PV+ interneurons (arrows) are located in all neocortical layers except L1. A dark axonal band delineates L4. The area in the black box is magnified in A1. Note the dense PV+ axonal staining and the large number of cell bodies. A2) The typical axo‐axonic (top, arrowheads) and basket (bottom, small arrows) axonal structures are visible in the acute samples. Axo‐axonic formations were the most prominently visible in L5/6. (B) The number of PV+ neurons has significantly decreased in D14 cultures, in all layers. The laminar structure of the neocortex is still visible; the layers can be distinguished. Note that only scattered PV+ interneurons are visible in L2/3 and L5/6. In L4, the loss of PV+ cells occurred in patches; the boxed area is magnified in B1. The neighboring area marked with an asterisk is lacking PV+ cells, while the axonal band is still visible. (B1) At D14, several cells with a considerably large cell body and long and numerous dendrites appear (upper cell marked with an arrow). (B2) The typical axo‐axonic (top, arrowheads) and basket (bottom, small arrows) formations are present in L5/6. (C) The number of PV+ interneurons has dramatically decreased at the end of the culturing period. In this sample from a D35 culture, cell bodies were present only in patches, mainly in L4. The axonal band still delineates L4, but the borders of other layers cannot be distinguished. The axonal band is present even in regions without PV+ cell bodies (asterisk), although less darkly stained than at earlier stages of culture. (C1) The box on the upper image is magnified. The remaining cells usually have a large cell body and numerous tortuous dendrites (arrows). (C2) The axo‐axonic (top, arrowheads) and basket formations (bottom, small arrows) are still present. (D) PV+ cell density was reduced during the culture period. The number of PV+ neurons dropped at the beginning, and then stayed low during the later period of culturing. The PV+ neuron density was significantly lower at D14 than at D0. (Kruskal‐Wallis ANOVA, ^*^ *p* = 0.0283, n(acute) = 14, n(D7) = 6, n(D14) = 8, n(D21) = 4, n(D28) = 3, n(30‐35) = 7, n(37‐42) = 2. (E) The use of the different cell culture medium had a significant effect on the PV+ cell density. It was significantly higher in artificial medium (AM) than in human cerebrospinal fluid (hCSF). (Mann‐Whitney test, ^***^ *p* = 0.0005, n(AM = 13, n(CSF) = 11). (F) Viral treatment had a significant effect on the PV+ cell density, which was significantly lower in virus‐treated samples compared to the non‐treated control. (Mann‐Whitney test, ^*^ *p* = 0.0448, n(control) = 32, n(virus) = 16).

Parvalbumin‐stained neurons are known to be perisomatic inhibitory interneurons in the human neocortex [38], comprising basket and axo‐axonic cells [39, 40]. Light microscopic analysis was done in 59 organotypic and 14 acute (D0) slices stained against PV, derived from 20 patients. In most of the acute postoperative tissue samples, numerous PV+ cells were visible in neocortical layers 2–6, with a dense axonal network in layer 4 (Figure 6A). The multipolar cells usually possessed long, well‐stained dendrites, oriented mainly perpendicular to the pial surface. The number of PV+ interneurons was reduced in organotypic cultures, already at D7, and remained very low throughout the culture period. Usually, in samples derived from epileptic patients PV+ cells remained visible for a longer period than in tissues from tumor patients. PV+ cells in L4 were preserved for the longest culture period and remained visible even at D35‐D42. At the beginning of the culture period (first two‐three weeks), the majority of the surviving PV+ cells showed similar morphology to the acute tissue (D0), but cells with large cell bodies and numerous long dendrites also appeared (Figure 6B). From two to three weeks in culture, we observed regions in the slices that lacked PV+ cell bodies, together with preserved axonal clouds (Figure 6B,C). At the end of the culture period (fourth to seventh week), the number of PV+ cells dropped close to zero, and large areas were void of stained elements. In these cases, the neocortical layers were not distinguishable (Figure 6C). The remaining cells were usually located in L4 and possessed large cell bodies and distorted dendrites. The axon terminal structures typical of axo‐axonic cells (the so‐called “chandeliers”), as well as the typical basket‐like formations characteristic of basket cells, were visible all over the culture period (Figure 6A1‐C2).

The change in PV+ cell density was quantified in acute and cultured slices (see Methods, Table 1; Table S9). The origin of the tissue did not impact the cell density in samples without viral injections (Kruskal‐Wallis ANOVA, *p* = 0.5206), and there was no significant difference between epileptic and non‐epileptic tissues at D0 (unpaired *t*‐test, *p* = 0.4912). Among the different culturing conditions, the medium type and the viral treatment caused significant differences (hCSF vs. AM: Mann‐Whitney test, *p* = 0.0005; viral treatment vs. control: Mann‐Whitney test, *p* = 0.0448); the presence of the antimycotic agent did not affect the cell density during culturing in hCSF (PS vs. PSA, Mann‐Whitney test, *p* = 0.0697). Considering the change in cell density across all measured samples over the whole culturing period (D0 to D42), the difference was significant (Kruskal‐Wallis ANOVA, *p* = 0.0194), as in the case of the week‐by‐week comparison (Kruskal‐Wallis ANOVA, *n* = 44 slices, *p* = 0.0082). The most remarkable difference was between the D0 and D14 states (post‐hoc test: *p* = 0.0283). When examining the day‐by‐day changes only in cultures without viral injection (from D0 to D42), we found no significant differences in the PV+ cell density (Kruskal‐Wallis test, *p* = 0.1044).

#### 2.4.5 Loss of Calretinin‐Stained Neurons

**FIGURE 7:**
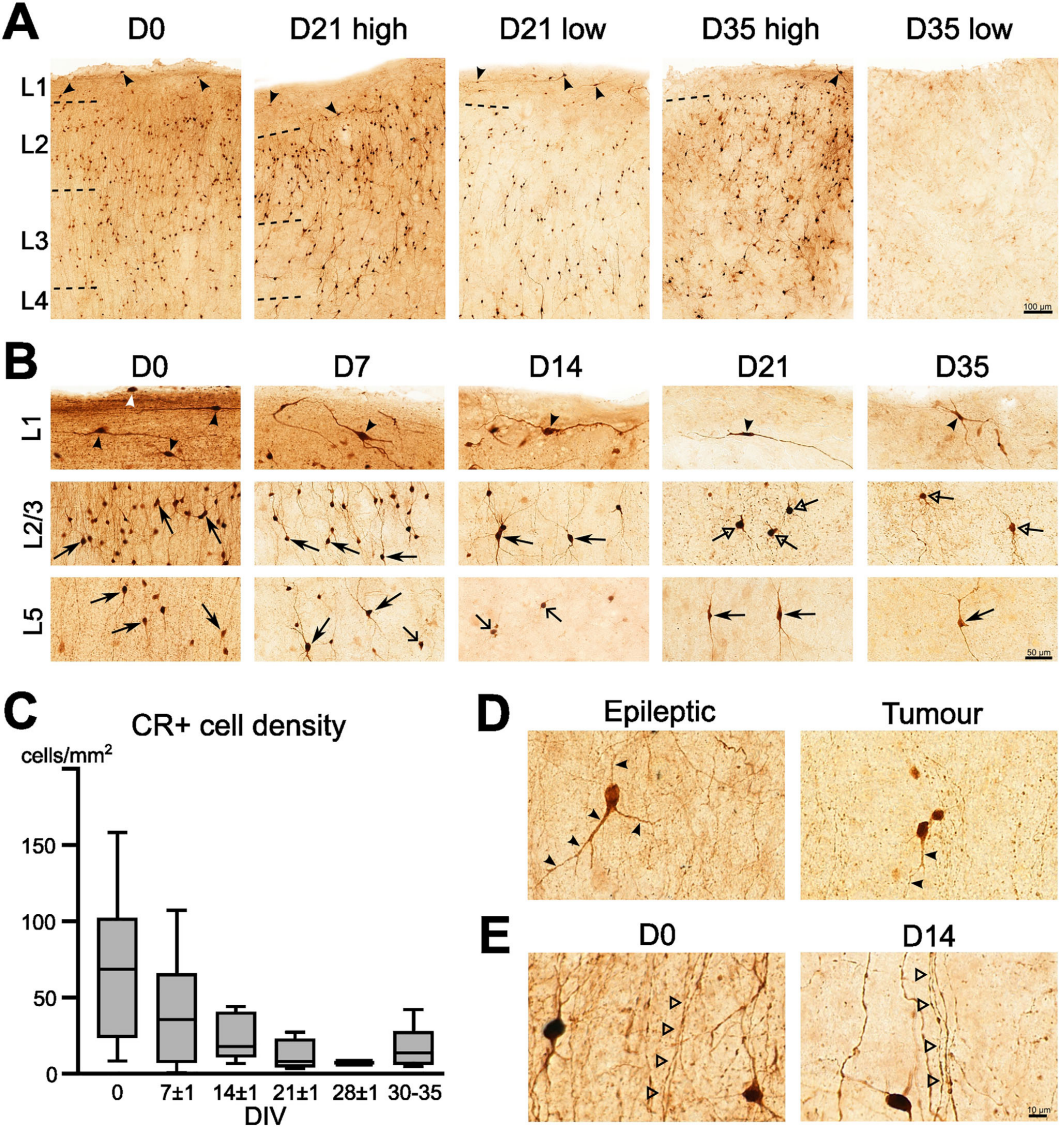
The number of CR+ cells considerably decreases during the culturing period. (A) Calretinin‐stained sections from acute sample (D0) and organotypic cultures (D21, D35). Numerous CR+ inhibitory interneurons are present in the human neocortex. Large horizontal bipolar cells are identified as Cajal‐Retzius cells in layer 1 (arrowheads, L1). CR‐stained neurons with high and low density are shown from organotypic slices fixed at D21 and D35. The images showing D21 are from different slices but from the same HOC sample (HOC25), whereas the images representing D35 are from the same slice. The neocortical layers cannot be separated at the later stages of the culture period. (B) Images show CR+ neurons in the different layers of the human neocortex at different time points during the culturing period. Cajal‐Retzius cells (arrowheads) are present in L1, from D0 to D35. Note the large cell body and the horizontal dendrites. Numerous cells are visible in L2/3 and L5 at D0, whereas their density decreases with culturing time. Arrows point to typical bipolar and multipolar cells, open arrows show the CR+ neurons with small cell bodies and very short dendrites, and open triangle arrows point to CR+ cells with distorted dendrites. These latter cells were usually seen at the later stages of the culture period. (C) The density of CR+ neurons from D0 to D35 is decreasing. Statistical tests did not show significant differences between the examined days in the week‐by‐week analysis. (n(D0) = 11, n(D7) = 6, n(D14) = 8, n(D21) = 4, n(D28) = 2, n(30‐35) = 6) (D) CR‐positive inhibitory cells usually had a more complex dendritic tree in epileptic (left image) than in non‐epileptic tumor (right image) tissue. Arrowheads point to the dendrites of the CR+ neuron. (E) Parallelly running CR+ dendrites were frequently seen in acute samples (D0), as well as at the earlier stages of the culturing time. Open triangle arrows point to the dendrite bundles.

Calretinin‐positive inhibitory neurons in the human neocortex are known to be either inhibitory interneurons found in each layer of the neocortex [41], or Cajal‐Retzius cells located in layer 1 [42]. To assess modifications in CR+ cells, we analyzed CR‐stained sections from 43 organotypic slices and 11 acute samples spanning D0‐D42 obtained from 11 patients. In the acute samples (at D0), numerous immunostained cells were visible in all layers of the neocortex. In layer 1, large bipolar horizontal cells were present, which were identified as Cajal‐Retzius cells (Figure 7A,B). In the other layers (L2‐L6), bipolar and multipolar cells with considerably smaller cell bodies were observed. These cells were usually perpendicularly oriented to the pial surface, and frequently possessed parallelly running dendritic bundles (Figure 7D). Calretinin‐positive cells in epileptic tissue usually had a more complex dendritic tree than cells in tumor tissue, and they preserved this feature throughout the culturing period. Beaded dendrites were observed in several cases, even in the acute samples. During the culturing period, the number of CR+ cells gradually decreased. However, the decrease was not homogeneous; after the second week of culture, slices with high and low numbers of CR+ interneurons were seen, sometimes derived from the same HOC sample, or patches of high cell density and empty areas in the same slice (Figure 7A). At the late stages of the culturing period (after D28), organotypic slices usually lacked CR+ cells; immunostained neurons were mainly present in patches (Figure 7A). Cajal‐Retzius cells were preserved throughout the culturing period, even in slices fixed at D35. The morphology of CR+ interneurons has also changed in the organotypic cultures. Cells with small somata usually disappeared earlier than neurons with a large cell body. Furthermore, CR+ neurons with no or very short dendrites were also frequently observed. In the second half of the culturing period (at D21‐42), cells with beaded or distorted dendrites were also often detected.

Viral treatment affected both the cell density and the morphology of the CR+ neurons. In most cases, the number of cells decreased considerably in virus‐injected slices compared to non‐injected slices from the same HOC sample. In virally treated slices, we found large areas lacking CR+ neurons, and few spots with CR+ cells that possessed either very short and/or distorted, beaded dendrites. Similar observations were made when comparing slices kept in hCSF vs. AM. Fewer cells were visible in hCSF than in AM, and they had more pathological morphology. Larger areas devoid of CR+ neurons were more typical of hCSF and virally treated slices than of AM or non‐injected ones.

To assess the changes in the cell density of CR+ interneurons, we performed semi‐automatic cell counting (see Methods) on the above‐mentioned 43+11 slices (for values see Table 1; Table S9). Although the morphology of the CR+ cells in the epileptic and tumor tissue was usually different, we did not find significant differences between epileptic and tumor patients (Mann‐Whitney test, *p* = 0.1143); therefore, these samples were pooled. No significant differences were observed when comparing tissues derived from different patients (HOC comparison, Kruskal‐Wallis ANOVA, *p* = 0.2865). The density of CR+ cells did not differ when examined with a day‐by‐day analysis (Kruskal‐Wallis ANOVA, *p* = 0.1743). Despite the visible decrease in cell density over the culturing period, the week‐by‐week changes were significantly different only in general (all data pooled together, ANOVA, *p* = 0.0182), but the post‐hoc test did not show any significant difference between the examined periods. Despite the morphological changes and the visible reduction in cell density across different media and viral treatments, neither affected cell density significantly (hCSF vs. AM: Mann‐Whitney test, *p* = 0.1624; viral treatment vs. control in AM: paired *t*‐test, *p* = 0.3447).

### 2.5 Effect of Tissue Source and Experimental Conditions on the Histological Outcome

Because tissues originated from different brain regions from patients with different ages, gender, and pathology, and because culture conditions varied across samples, we performed multivariate Linear Mixed Effects (LME) analysis to evaluate the influence of the tissue source and other key experimental covariates on histological, electrophysiological, and calcium imaging outcomes. Patient identity was included as a random effect, and the evaluated fixed effects included cortical region, age, gender, pathology, viral treatment, culture medium, and antimicrobial treatment. Histological outcomes comprised NeuN density, GFAP coverage, IBA1 coverage, PV cell density, and CR cell density. Model specifications, sample sizes, effect estimates, and exact *p*‐values are reported in the Supporting Information, including Tables S10 and S11. The tissue source and the experimental conditions did not considerably influence the histological outcomes, except for several effects. Pathology (i.e., epilepsy or tumor) influenced NeuN density and IBA1 coverage; lobe of origin had an effect on PV density, whereas gender had a marginal effect on CR density. These findings should be considered cautiously because the distributions of patient characteristics and experimental conditions were sparse, unbalanced, and partly confounded. The available electrophysiological and calcium‐imaging datasets contained too few patients to support comparable multivariable models (*n* = 4 and *n* = 3, respectively). Consequently, the effects of patient characteristics and experimental conditions on functional outcomes could not be reliably estimated.

### 2.6 Relationship Between Functional and Structural Observations

Slices used for electrophysiological or calcium‐imaging experiments were fixed after recording and included in the histological analysis where tissue quality permitted. However, the modalities differed in sampling design: calcium imaging followed a limited number of slices longitudinally, whereas electrophysiology and histology primarily sampled different slices across culture ages. The study was therefore not designed to estimate direct correlations between individual functional and histological variables. At the group level, spontaneous activity remained detectable and selected activity measures stabilized during the same culture period in which NeuN‐positive neuronal density declined, laminar organization deteriorated, interneuron‐marker densities decreased, and glial morphology changed. These observations demonstrate divergent functional and structural trajectories but do not establish a causal relationship between them.

## 3 Discussion

### 3.1 Functional Reorganization and Stabilization of Adult Human Cortical Networks In Vitro

Human organotypic slice cultures retained robust spontaneous neuronal activity throughout the culture period, as demonstrated independently by extracellular electrophysiology and longitudinal calcium imaging. Instead of indicating a simple preservation of the native network [18, 25, 43], our results suggest that human organotypic brain slices possess a remarkable ability to adapt to long‐term culturing, where an extensive structural reorganisation is linked to the emergence of stable neuronal activity similar to that observed in the acute slices. The spontaneous population events observed in acute and cultured slices lacked the hallmarks of epileptiform discharges reported in organotypic cultures under pathological conditions, namely large‐amplitude stereotyped bursts with near‐complete neuronal recruitment and prolonged hypersynchronous discharges [25, 44]. In contrast, the events detected in the present study occurred within physiological firing range and displayed heterogeneous recruitment across neuronal classes, consistent with coordinated population dynamics rather than pathological hypersynchrony [15, 34].

The recorded neuronal population also retained a physiologically plausible cellular composition across patients and time points. Approximately 75–80% of isolated units were classified as principal cells, closely matching the excitatory‐to‐inhibitory neuronal ratios described in adult human neocortex [45] and reported in previous human organotypic preparations [26, 30]. Reliable separation of interneurons and principal cells based on spike waveform further confirms that canonical electrophysiological cell type identities are retained in culture [46] and principal neurons remain the dominant contributors of spontaneous network activity in vitro [15, 47].

Spike waveform dynamics revealed a significant narrowing of action potential half‐width in principal cells over the culture period, while interneuron waveforms remained comparatively stable. Experimental and modeling studies indicate that activity‐dependent maturation processes can modulate sodium channel density and spike initiation dynamics, leading to sharper action potentials and improved temporal precision [48, 49]. Because fast‐spiking interneurons already operate with optimized spike kinetics, their extracellular waveforms may be less susceptible to such plasticity. The selective evolution of pyramidal‐cell spike kinetics therefore likely reflects adaptive intrinsic plasticity during circuit reorganization in vitro.

Several electrophysiological metrics followed coherent temporal trajectories during the culture period. Burst generation in principal cells increased, and discharge irregularity (ISI CV) transiently increased during the intermediate culture phase (D7–15) before returning toward baseline values by D22. These changes closely resemble the sequence described after traumatic deafferentation and axotomy: acute loss of long‐range inputs, transient hyperexcitability associated with reactive synaptogenesis and altered excitation‐inhibition balance, followed by homeostatic stabilization through intrinsic and synaptic plasticity [50, 51]. Comparable dynamics have been reported in both rodent and human slice preparations, where early circuit perturbations are gradually compensated by intrinsic and synaptic homeostatic mechanisms [23, 51].

Analysis of single‐unit recruitment during spontaneous population activity events further revealed distinct cell‐type‐specific participation patterns. Intrinsically bursting principal cells were the most consistently recruited neuronal class (93.75%), supporting that burst‐capable pyramidal neurons contribute disproportionately to the generation of synchronous population activity [15, 52]. Interneurons were recruited less frequently overall, but those that participated in population events exhibited approximately twofold higher firing rates than non‐recruited interneurons, suggesting that strongly driven and highly active inhibitory neurons also participate in the generation and maintenance of population activity. This is consistent with the rapid engagement of fast‐spiking interneurons during synchronized cortical activity and likely contributes to temporal coordination rather than initiation of synchronous events [26, 53].

SPA properties showed no statistically significant change across recording days, indicating that the capacity for generating population activity is maintained throughout the culture period. However, several electrophysiological features evolved coherently over culturing time. Multiple‐unit activity and single‐unit level features such as discharge regularity (ISI CV) and the proportion of recruited neurons reflected a transient perturbation during the early culture phase (D7‐15), then returned toward D0 values by D22. Importantly, this progressive convergence of electrophysiological properties was consistent, despite the substantial inter‐patient variability. These findings suggest that, following an early phase of network disturbance – presumably linked to the considerable neuron loss – human cortical organotypic cultures reach a state by around three weeks in which single‐unit firing patterns and population‐event recruitment more closely resemble those of the acute tissue. This contrasts with the progressive development reported in rodent organotypic cultures, where developmental stage and genetic background of the original brain tissue strongly influence network maturation [54]. In human preparations using adult tissue, despite the highly diverse clinical background and tissue origins, intrinsic circuit dynamics reorganize toward reproducible neuronal activity patterns during prolonged in vitro maintenance [28]. Our findings extend this observation by demonstrating that both single‐unit firing and population recruitment patterns stabilize within approximately three weeks, defining a reproducible window for experimental functional investigation.

Wide‐field Ca^2+^‐imaging provided complementary evidence for functional stabilization by following the same slices repeatedly over the culturing period. Recurrent population Ca^2^^+^ transients were detected in line with the development of GCaMP expression, which are most probably the optical readouts of electrophysiologically recorded SPAs. This activity persisted for weeks, forming spatially confined and temporally stable activity hubs. These active regions – mainly expanding to both supragranular and infragranular layers – were consistently observed across repeated recordings, indicating that network organization is not random but constrained by underlying structural connectivity. The dimension and the temporal evolution of the active area were closely related to the GCaMP expression in almost all slices. Recurrent synchronous Ca^2+^‐transients indicate that coordinated population activity remains detectable in culture. Similar stable activity patterns have been reported in human hippocampal slice cultures and are thought to reflect preserved microcircuit motifs [28, 29]. The persistence of these population activities, together with the convergence of single‐unit dynamics, suggests that network‐level attractor states emerge despite ongoing neuronal loss and structural remodeling.

In contrast to linear electrophysiology, wide‐field imaging – by the readout of the entire slice – showed that all virus‐transduced cultured slices generated calcium transients most probably corresponding to synchronous population events. These findings may be explained, at least in part, by GCaMP expression, which has been shown to alter neuronal excitability and shift neurons toward a more active state [55]. Moreover, viral transduction was accompanied by a reduced density of inhibitory interneurons, suggesting that the resulting imbalance between excitation and inhibition also facilitates the generation of synchronous activities.

### 3.2 Structural Reorganization Associated with Neuronal Activity Stabilization

Histological analysis of the slices generating cellular and population activities revealed substantial structural reorganization in human cortical organotypic cultures. As in rodent organotypic cultures [56], neuronal density decreased significantly in our human slice cultures, particularly during the first week, accompanied by the partial degradation of the laminar architecture [24, 57]. Similar early neuronal loss has been described in rodent and human organotypic cultures and is widely considered an inevitable consequence of tissue slicing, axotomy, and metabolic stress [20]. Neuronal loss was very variable across different samples, but also across temporal and spatial dimensions. Remarkably, different neuronal densities could be detected at the same time point of the culturing period, as well as in different slices from the same sample (even on the same day in vitro). Neuron loss could affect the entire neocortex or be confined to specific layers or a specific area, especially related to viral injection. This spatial/temporal variability might be related to the diversity of the patients and thus might be a human‐specific feature. However, operation and slicing circumstances can also affect diverse neuronal survival [30].

Inhibitory interneuron populations were particularly vulnerable. The loss of parvalbumin‐ and calretinin‐positive interneuron types is consistent with prior reports highlighting the susceptibility of inhibitory neurons to stress, such as human epilepsy [58, 59] or ischemia [60]. However, the surviving parvalbumin‐ and calretinin‐positive cells preserved their functional identity: both types of perisomatic interneurons (PV+ axo‐axonic and basket cells) as well as CR+ Cajal‐Retzius cells were observed even at the latest stages of the culturing period. The survival of Cajal‐Retzius cells is in contrast with findings in mouse organotypic cultures, where this cell type—together with all CR+ inhibitory cells—is totally lost during the first week [61]. However, this might be a human‐specific feature, as unlike in rodents [62], Cajal‐Retzius cells persist in the adult human neocortex [63]. The extensive reduction of these interneurons represented one of the most striking structural findings in our study, suggesting an increased excitatory/inhibitory neuron ratio over the culturing period. However, as the examined inhibitory cell types constitute only a small subset of all neocortical interneurons [64], and parvalbumin is known to disappear from the cells in response to stress [65, 66], one should treat this conclusion with caution. Furthermore, the ratio of our clustered interneurons also indicates that interneuron loss is not dramatically higher than the overall neuronal death. Inhibitory interneurons are key regulators of cortical excitability, spike timing, and network synchronization [67, 68], and their selective vulnerability, however, might contribute to the transient increase in principal‐cell burst propensity and discharge irregularity observed during the first two weeks in vitro. Nevertheless, electrophysiological activity returned toward acute baseline values by the fourth week despite the remarkable interneuron loss, suggesting that compensatory mechanisms emerge within the remaining local circuitry. This dissociation between structural deterioration and functional stabilization illustrates the remarkable adaptive capacity of adult human cortical networks.

Glial responses were prominent, quickly developed, and persisted until the end of the culturing period. Similar to the neuronal loss, the degree of both astroglial and microglial coverage was variable; patches containing a sparse glial network intermingled with highly covered areas. Astrocytes exhibited a pronounced reactive gliosis, particularly at the slice surfaces, consistent with injury‐induced astrocytic reorganization (for review, see [69]). Microglial cells displayed diverse activation states, transitioning from homeostatic to reactive and phagocytic phenotypes, in line with their roles in synaptic remodeling and debris clearance [70]. Notably, while glial morphology changed dramatically, the overall coverage remained relatively stable, suggesting that qualitative rather than quantitative changes in glial function accompany network reorganization.

### 3.3 Translational Implications and Limitations

In contrast to previous findings [18, 23, 30], cultures maintained in artificial medium showed better morphological preservation than those maintained in hCSF under our experimental conditions. Lower neuronal densities and lower astroglial coverage suggest a more pronounced overall cell loss in hCSF. However, hCSF collection and quality were suboptimal in our experiments; hCSF was not standardized, and its biochemical composition was not systematically characterized. Our findings therefore should not be generalized to well‐characterized hCSF preparations or interpreted as evidence that artificial medium is intrinsically superior. Rather, they indicate that an appropriate artificial medium [24] maintained cultures for up to 42 days more consistently than the variable hCSF preparations.

Our findings indicate that adult human organotypic cultures should be viewed as a model of post‐injury adaptive circuit reorganization rather than static preservation of the native cortex. Surgical resection and slice preparation inevitably damage long‐range afferent and efferent projections, disrupt vascular perfusion, induce local axonal and dendritic injury, and modify neuron‐glia interactions [71, 72]. These preparations therefore cannot reproduce systems‐level brain function or higher‐order cortical computations. Nevertheless, they provide experimental access to mature human cortical tissue with retained aspects of its cellular composition and local architecture. Importantly, the persistence of cellular and population activity in culture does not establish preservation of the original microcircuit; it may instead reflect an adaptive stabilization within a structurally altered network.

An additional limitation is that functional characterization relied primarily on spontaneous activity. Although spontaneous firing, population events, and calcium dynamics provide sensitive measures of circuit viability and network organization, they do not directly establish preservation of cortical computations such as stimulus‐response transformations or synaptic information transfer. In addition, calcium imaging and electrophysiology were not acquired simultaneously, and the present analyses do not directly identify the mechanisms underlying functional stabilization. Future studies combining electrical or optogenetic stimulation, high‐density electrophysiology, functional connectivity analyses, and perturbation experiments will therefore be important to determine how faithfully adult human organotypic cultures preserve computational properties of cortical networks.

Overall, adult human organotypic cultures exhibited substantial structural alterations after surgical resection and slicing, accompanied by an early period of functional variability and subsequent stabilization of selected measures of spontaneous activity. Although this temporal pattern is consistent with compensatory or homeostatic reorganization, the underlying mechanisms were not directly tested. The electrophysiological, calcium‐imaging, and histological findings therefore support adult human organotypic cortical cultures as a complementary model for studying local spontaneous activity and structural transformation in mature human tissue. Their pathological tissue origin, patient heterogeneity, limited scalability, loss of long‐range connectivity, vascular support and immune reactions, as well as culture‐induced remodeling constrain generalization to the intact human cortex.

## 4 Conclusion

Conceptually, our findings indicate that substantial structural degeneration and functional stabilization are not opposing processes but co‐emergent features of network adaptation. While neuronal loss, gliosis, and interneuron vulnerability reshape the anatomical organization, the temporal pattern of the functional read‐outs is consistent with homeostatic or compensatory mechanisms, which drive the network toward stable attractor states that preserve coherent activity patterns. The combination of electrophysiology, longitudinal calcium imaging, and detailed quantitative histology demonstrates that human organotypic cultures do not simply deteriorate over time but instead can continue generating spontaneous neuronal activity despite the profound structural deterioration. Human slice cultures advance from an early perturbed state toward a reorganized system in which spontaneous population synchrony, cell‐type‐specific firing patterns, and recruitment dynamics are maintained. These findings support adult human organotypic cultures as a complementary experimental model for studying local spontaneous activity and injury‐driven structural remodeling in mature human cortical tissue under conditions inaccessible in vivo.

## 5 Methods

### 5.1 Patients

The patients were operated at the Clinic for Neurosurgery and Neurointervention, Semmelweis University, 1145 Budapest, Hungary. We received written consent from all patients. Our protocol was approved by the Medical Research Council of the Ministry of Interior (ETT TUKEB, IV‐8358/3/2021/EKU and IV‐8360/3/2021/EKU) and performed in accordance with the Declaration of Helsinki. Neocortical tissue samples were resected from 30 patients (10 females, 20 males; Table S1). We obtained tissue from frontal (*n* = 5 patients), parietal (*n* = 2), and temporal (*n* = 23) lobes. The patients suffered from epilepsy and/or tumor. In the case of tumor patients, the resected tissue was always outside of the tumor area.

### 5.2 Tissue Preparation and Culturing

Tissue was transported from the operating room to the laboratory and processed for culturing in an ice‐cold, oxygenated NMDG solution [24]. Neocortical slices of 350 µm thickness were cut with a Leica VT1200S vibratome (Leica Biosystems, Buffalo Grove, IL, USA, RRID:SCR_016495). In total, 297 organotypic cultured slices were made from samples obtained from 30 patients; on average, *n* = 10±6 slices were cultured, ranging from 3 to 24 per brain sample. Slice cultures made from one patient were named as HOC (human organotypic culture). Samples obtained from 18 patients were cultured in artificial medium (AM), supplemented with penicillin, streptomycin, and amphotericin B (PSA), modified from [24]. Slice cultures made from *n* = 12 samples were maintained in human cerebrospinal fluid (hCSF), including 7 cultures with only PS and 5 cultures with PSA supplement (for details see Supporting Information). Cultured slices were fixed weekly for post hoc anatomical investigations, regardless of culture size (i.e., number of slices), to continuously visualize architectural modifications in the neocortex. Furthermore, the preservation of the cultured tissues was screened on a daily basis. If signs of low‐level preservation (uneven flattening of the slice, disintegration of the grey matter, low adhesion capacity to the insert) were visible, the slice was immediately fixed, breaking the week‐by‐week fixation rule. With these aspects in mind, we intended to maintain each culture of the highest quality achievable as long as its size allowed.

### 5.3 Virus Injection

Adeno‐associated viruses (AAVs) PHP.eB‐EF1a‐GCaMP6s‐WPRE‐pGHpA, (titer: 2.5 × 10^13^ vg/mL, Addgene #67526), PHP.eB‐Syn‐jGCaMP7s‐WPRE (titer: 2.8 × 10^14^) vg/mL, Addgene #104487) were injected into *n* = 42 slices (derived from 10 patients). At D0, 10 µL was administered into the tissue with a Hamilton‐pipette, either at full concentration or diluted to 1:10 or 1:100 in 1xPBS. Slice cultures injected with viruses were distinguished from non‐injected slices and were considered a different HOC sample.

### 5.4 Recordings

Both acute slices and organotypic cultures were transferred and maintained at 35–37°C in an interface chamber, perfused with a standard physiological solution, and the extracellular local field potential gradient (LFPg) recording was obtained as described previously [34] (see Supporting Information).

Wide‐field imaging was used to assess the calcium (Ca^2+^) signal of GCaMP‐positive neurons. Fluorescence imaging was performed on a custom wide‐field mesoscope equipped with a tandem lens configuration, utilizing a 50 mm f/1.4 objective lens (Nikon) and a 50 mm f/0.95 imaging lens (Navitar) to yield an effective magnification of 1×. Excitation light from a 470 nm LED (M470L4, Thorlabs) was passed through a 472/30 nm bandpass filter (FF02‐472/30‐25, Semrock) and coupled into the optical path via a 495 nm dichroic mirror (T495lpxr, Chroma). Emitted fluorescence was collected through a 525/50 nm band‐pass emission filter (86‐963, Edmund Optics) and detected by a scientific CMOS camera (Prime BSI Express, Teledyne Photometrics). Image acquisition was coordinated using custom Python software interfacing with the camera's PVCAM library via the PyVCAM wrapper.

### 5.5 Data Analysis

Electrophysiology data were analyzed with the Neuroscan Edit4.5 program (Compumedics Neuroscan, Charlotte, NC, USA), and home‐written routines for Python 3.14 (Python Programming Language, RRID:SCR_008394).

Detection and analysis of spontaneous population activity (SPA) were performed as described previously [34] (for details see Supporting Information). Briefly, SPA peaks were detected after a manually implemented thresholding on the channel which showed the largest amplitude SPA. The detected events were manually verified and corrected for artefacts, if necessary.

Action potentials (APs) were clustered using a multi‐stage pipeline. Initially, spike detection was performed using Kilosort4 [73] with a lowered detection threshold (set to 5) to maximize the capture of low‐amplitude units. Initial cluster curation was conducted via the Phy interface (https://github.com/cortex‐lab/phy); however, due to the lower signal‐to‐noise ratio inherent in long‐cultured slices, these outputs were insufficient for high‐fidelity isolation. Consequently, we implemented a custom‐written Python post‐processing framework that applies user‐defined, data‐inspection‐based thresholding and rigorous waveform stability criteria to refine these clusters (code available upon request). Based on their AP width and firing characteristics, inhibitory interneurons (IN) and excitatory principal cells (PC), further divided into intrinsically bursting (IB) and regular spiking (RS) types, were separated [15].

Analysis of single‐cell firing was investigated with the home‐written program in Python. For each cell, average firing frequency, inter‐spike‐interval coefficient of variation (ISI CV), and two measures for burstiness were calculated. When a set of three APs was detected within 20 ms, they were considered to be part of a potential burst. Bursts containing more than three APs could be longer than 20 ms, but each group of three consecutive APs had to lie within a 20 ms period. Burstiness index 1 was determined as the ratio of APs within bursts, whereas burstiness index 2 was the percentage of APs within 20 ms on the autocorrelogram. We also investigated the potential increase in firing rate of each cell compared to SPA events (within ±50 ms of the SPA peak) using an adapted Monte Carlo approach [34].

Wide‐field fluorescence recordings were processed to extract whole‐slice and manually defined regional Ca^2+^‐transient rates. Following initial quality control to verify temporal consistency, a time‐collapsed mean image was generated for each recording, followed by additional image processing steps to improve signal contrast (see Supporting Information). Ca^2+^‐transients were automatically identified in the corrected signal using amplitude, prominence, and minimum‐distance constraints. Finally, the mean Ca^2+^‐transient rate (Hz) was calculated by dividing the total detected peak count by the recording duration.

The activation maps were calculated by pixel‐wise multiplication of a standard deviation map and a local correlation map. The local correlation map was calculated as described and implemented in the CaImAn toolbox [74]. The two maps were percentile‐normalized between the first and 99th percentile range before multiplication.

Anatomical identification of the cortical layers for GCaMP expression and activity included analysis of native images of the slices, together with the time‐collapsed mean image, and post hoc NeuN‐, GFAP‐ or PV‐stained sections of the same slice, if available. If post hoc anatomical sections could not be used, layer borders were estimated from neighboring slices (Figure S3). Supragranular (L1‐3), granular (L4), infragranular (L5‐6) layers and the white matter were delineated.

### 5.6 Histology

Immunostaining was performed on fixed slice cultures to visualize neuronal and glial presence and morphology, as well as to verify the laminar structure of the neocortex and the cultured slices, preferably in a week‐by‐week screening. Acute and cultured slices used for electrophysiological or wide‐field recordings were also fixed after the experiment with a fixative containing 4% paraformaldehyde in 0.1 m phosphate buffer (PB), applied for 24 h. 60 µm thick sections were made from the slices with a Leica VT1200S vibratome. Sections were immunostained against the neuronal cell body marker NeuN, the astroglial marker glial fibrillary acidic protein (GFAP), the microglial marker IBA1, the inhibitory cell markers parvalbumin (PV), calretinin (CR), or the green fluorescent protein for viral expression detection (GFP). For the exact protocol, see Supporting Information.

All cultured slices were resected, usually into 5 to 10 sections of 60 µm thickness. Generally, all sections made from cultured and acute slices were processed for immunostaining. Sections were excluded if (i) they did not contain any grey matter, only white matter, (ii) they fell apart into small pieces during the immunostaining‐mounting process. The sorting strategy for the different stainings was as follows: NeuN and GFAP staining were performed on all slices, using sections containing all six neocortical layers. The sections containing the majority of the layers were assigned to the interneuronal stains and IBA1. The remaining sections (usually at the edge/surface of the cultured slice) were added to either NeuN or glial staining to investigate the differences within the cultured slice.

### 5.7 Quantitative Morphological Analyses

In the quantitative analysis, all automatically scanned and well‐focused sections were included. Folded sections or areas were excluded. We determined neuronal density in NeuN‐, PV‐, and CR‐stained sections and both astroglial and microglial coverage in GFAP‐ and IBA1‐stained sections, respectively, in slices fixed at different time points, with the aid of the QuPath v0.6.0 software [75, 76] in batch processing. The general tissue detection was corrected by hand in the case of the neuronal cell densities, limiting it to the grey matter, as well as in the case of the IBA1 perimeter‐to‐area ratio, limited to a representative rectangle within the section. To assess possible statistical differences, we averaged data obtained at D7±1 (week1), D14±1 (week2), D21±1 (week3), D28±1 (week4), and D32 to D45 (week5). For details, see Supporting Information.

### 5.8 Statistical Analysis

If the data followed a normal distribution (verified with the Kolmogorov‐Smirnov & Shapiro‐Wilk tests), a t‐test or an unpaired t‐test was used to compare two groups. In the case of multiple groups, ANOVA (unpaired condition) or ANOVA with Greenhouse‐Geisser correction (paired condition) was performed. If the normality test failed, we used the Wilcoxon signed‐rank test (two groups, paired) and Mann‐Whitney U test (two groups, unpaired) or the Kruskal‐Wallis test (multiple groups, unpaired) and Friedman test (multiple groups, paired) for comparing two or multiple groups, respectively. When comparing multiple groups, Dunn's post‐hoc test with Bonferroni correction was used when the main test reached significance (*p* < 0.05). Significance thresholds were defined as: ^*^ *p* < 0.05, ^**^ *p* < 0.01, ^***^ *p* < 0.001. For quantitative histology, we determined statistical significance using GraphPad Prism version 10.0.0 for Windows (GraphPad Software, Boston, MA, USA, RRID:SCR_002798). Statistical analyses of electrophysiological data were performed in Python (v. 3.14) using a custom analysis pipeline, available upon request.

Event‐relatedness of each unit was tested via surrogate statistics [15], which either classified a unit as “increased” (significantly elevated firing during population events) or “indifferent”. Differences in the proportion of event‐related units across groups (cell types, days‐in‐vitro) were assessed using Pearson's chi‐squared test of independence, with results reported as χ^2^, degrees of freedom, and *p*‐value.

The histological data were grouped, filtered, and pre‐processed as described in the Quantitative Morphological Analyses section and Supporting Information. Briefly, due to the heterogeneity of the samples and the culturing protocols, outliers were not excluded; normalization for the cell densities was applied only if multiple sections were stained from the same cultured slice. The normalization was proportional to the size of the sections. All statistical analyses in the Results section were described as data presentation, sample size, and the applied statistical test. After normalization testing, two‐sided testing was performed as described above. For more details, see Supporting Information, Histology part.

### 5.9 Artificial Intelligence Generated Content Statement

For code refactoring and maintenance, a locally hosted instance of Qwen 3.6‑35B‑A3B was used. All outputs were manually verified and reformulated where necessary to ensure accuracy and scientific rigor. No artificial intelligence was used to generate any other parts of this work.

## Author Contributions

**Estilla Zsófia Tóth**: Conceptualization, Data curation, Investigation, Methodology, Resources, Validation. Made all organotypic cultures up to HOC29, led all immunostaining (including recutting the slices) and all wide‐field imaging. Led the scanning process of all stained sections. **Rebeka Stelcz**: Conceptualization, Data curation, Formal analysis, Investigation, Methodology, Resources, Software, Visualization, Writing – original draft. Helped with culturing from the beginning; continued it alone from HOC30. Participated in the recutting, staining, and scanning; led the entire quantitative histology part with statistics. Performed NeuN, GFAP, and IBA1 detection and quantitative analysis alone; led PV and CR cell counting. Wrote the paper and made figures. **Réka Bod**: Conceptualization, Data curation, Formal analysis, Methodology, Software, Writing – original draft. Developed the framework for the ephys analysis pipeline, wrote the SPA detection and clustering software. Clustered all single cells, analyzed the ephys SPA part, made all statistics for the ephys part. Participated in the human operations. Wrote the paper. **Ábel Petik**: Conceptualization, Data curation, Formal analysis, Investigation, Methodology, Software, Visualization. Developed the custom wide‐field imaging platform and software pipeline for longitudinal calcium imaging; performed and analyzed wide‐field imaging experiments. **Eszter Juhász**: Data curation, Formal analysis, Investigation, Resources. Cell counting (half‐manual) for PV and CR sections, participated in human tissue preparation, recutting and staining the slices, part of human data administration. **Kinga Tóth**: Conceptualization, Data curation, Investigation, Methodology, Project administration, Resources, Supervision, Validation, Writing – original draft. Led and performed most ephys experiments, led SPA detection, wrote the paper, did human tissue preparation, led and made the administration of human data. **Liza Szalai**: Data curation, Investigation: Recutting the slices, staining, scanning, and analysis of GFAP+ sections. **Nour Essam**: Investigation, Project administration. Performed ephys experiments, administered ephys experiments, and participated in human tissue preparation. **Fanni Somogyi**: Data curation, Investigation. Performed wide‐field imaging experiments. **Beatrix Kovács**: Methodology, Resources. Provided viral tools used for imaging experiments. **Amirmahdi Mojtahedzadeh**: Data curation. Performed most SPA detection. **Attila György Bagó**: Project administration, Resources: Neurosurgeon, provided human tissue. **Loránd Erőss**: Project administration, Resources, Neurosurgeon, provided human tissue. **Péter Orbay**: Project administration, Resources, Neurosurgeon, provided human tissue. **Johanna Petra Szabó**: Project administration, Resources, organized the operations, helped in providing clinical data. **Dániel Fabó**: Project administration, Resources, Neurologist, provided human clinical data. **Boglárka Hajnal**: Project administration, Resources, Neurologist, provided human clinical data. **Bence Rácz**: Resources, Supervision, Writing – review & editing. Provided scanning possibilities, taught scanning method, reviewed and edited the manuscript. **Daniel Hillier**: Conceptualization, Funding acquisition, Methodology, Resources, Supervision, Software, Writing – review & editing. Conceived the longitudinal wide‐field imaging approach, supervised the development of the imaging platform and associated analysis workflow, and contributed to the viral strategy/tools used for the imaging experiments; reviewed and edited the manuscript. **István Ulbert**: Conceptualization, Funding acquisition, Methodology, Project administration, Software, Writing – review & editing. Concept of ephys recordings and analysis (both SPA detection and clustering), supervision of the ephys pipeline development and analysis, wrote the ethical approval, reviewed and edited the manuscript. **Lucia Wittner**: Conceptualization, Data curation, Funding acquisition, Investigation, Methodology, Project administration, Resources, Supervision, Visualization, Writing – original draft. Concept of the overall study, coordination of the experimental workflow, led and participated in human tissue preparation, contributed to the ephys pipeline development, supervision of the entire histology and ephys parts, wrote the ethical approval, wrote the paper, made figures.

## Funding

This research was funded by the National Research, Development, and Innovation Office, grant nos. FK129120 (to K.T.), K137886, Advanced 150799 (to L.W.), Excellence 151368, CELSA/24/020 and KSZF 161/2024 (to D.H.); by the Hungarian Brain Research Program, grant no. NAP2022‐I‐2/2022 (to I.U.); by the János Bolyai Research Scholarship (to K.T.) and the Lendület (“Momentum”) Programme (to D.H.) of the Hungarian Academy of Sciences, and by the EU: FLAG‐ERA Joint Transnational Call 2021, VIPattract grant (to L.W.); 2019‐2.1.7‐ERA‐NET‐2021‐00047 (to D.H.). This work was supported by Project No. RRF‐2.3.1‐21‐2022‐00001 under the Recovery and Resilience Facility and was also conducted within the National Laboratory of Infectious Animal Diseases, Antimicrobial Resistance, Veterinary Public Health and Food Chain Safety, University of Veterinary Medicine Budapest (to B.R.). The scientific research and results published here were reached with the sponsorship of Gedeon Richter Talentum Foundation in the framework of Excellence PhD Scholarship of Gedeon Richter (to R.S. and to F.S.), with the Doctoral Student Scholarship Program of the Co‐operative Doctoral Program of the Ministry of Innovation and Technology (to R.B. and B.K.), and the Semmelweis 250+ Graduate Student Fellowship of the Semmelweis University (to R.S., R.B. and F.S.).

## Ethics Approvals

The presented study and protocol were approved by the Hungarian Medical Research Council of the Ministry of Interior (ETT TUKEB, IV‐8358/3/2021/EKU and IV‐8360/3/2021/EKU), and all procedures adhered to the principles of the Declaration of Helsinki.

## Conflicts of Interest

The authors declare no conflicts of interest.

## Supporting information




**Supporting File**: advs77795‐sup‐0001‐SuppMat.docx.

## Data Availability

The data that support the findings of this study are available on request from the corresponding author. The data are not publicly available due to privacy or ethical restrictions.
